# Pooled Analysis of Mesenchymal Stromal Cell-Derived Extracellular Vesicle Therapy for Liver Disease in Preclinical Models

**DOI:** 10.3390/jpm13030441

**Published:** 2023-02-28

**Authors:** Xinru Fang, Feiqiong Gao, Qigu Yao, Haoying Xu, Jiong Yu, Hongcui Cao, Shibo Li

**Affiliations:** 1 Department of Infectious Disease, Zhoushan Hospital, Zhejiang University School of Medicine, Zhoushan 316021, China; 2 State Key Laboratory for the Diagnosis and Treatment of Infectious Diseases, Collaborative Innovation Center for Diagnosis and Treatment of Infectious Diseases, The First Affiliated Hospital, Zhejiang University School of Medicine, Hangzhou 310003, China; 3 Department of Laboratory Medicine, The Fourth Affiliated Hospital, International Institutes of Medicine, Zhejiang University School of Medicine, Yiwu 310003, China; 4 Jinan Microecological Biomedicine Shandong Laboratory, Jinan 250117, China; 5 Key Laboratory of Diagnosis and Treatment of Aging and Physic-Chemical Injury Diseases of Zhejiang Province, 79 Qingchun Rd, Hangzhou 310003, China

**Keywords:** extracellular vesicle, mesenchymal stromal cell, liver disease, meta-analysis

## Abstract

Background: Although increasing preclinical studies have emphasized the benefits of exosome-related therapies, the efficacy of mesenchymal stromal cell (MSC)-derived extracellular vesicles (EV) for liver injury is unclear. In this work, a pooled analysis was conducted to explore the overall effect of MSC-EV in animal models. Methods: A systematic search of the PubMed, EMBASE, Web of Science, and Cochrane Library databases was performed, from initiation to February 2022, for preclinical studies with liver disease models. The treatment outcomes were evaluated based on liver function, histological analysis, and inflammatory cytokines. Results: After screening, 39 studies were included. Pooled analyses demonstrated that MSC-EV therapy significantly improved liver functions (ALB, ALT, AST, ALP, and γ-GT), promoted the repair of injured liver tissue (damaged area, Ishak’s score), reduced inflammatory factors (TNF-α, IL-1β, IL-6, and IFN-γ), and increased an anti-inflammatory cytokine (IL-10) compared to the placebo control group. Subgroup analyses indicated that MSC-EV had therapeutic effects on liver fibrosis (n = 16), acute liver injury (n = 11), non-alcoholic fatty liver disease (n = 3), autoimmune hepatitis (n = 4), and hepatic ischemia-reperfusion injury (n = 6). Additionally, the therapeutic effect of EV was comparable to that of MSCs. Conclusion: MSC-EV have therapeutic potential for acute and chronic liver diseases.

## 1. Introduction

In the past few decades, liver disease has been on the rise, and it is a major cause of death and illness worldwide [1]. The Global Burden of Disease 2010 study showed that the number of liver cirrhosis deaths worldwide increased from around 676,000 in 1980 to > 1 million in 2010, thus accounting for approximately 2% of all deaths worldwide [2]. China is also experiencing a surge in liver disease. It is estimated that over one-fifth of the population of China is affected by some form of liver disease [3]. Based on epidemiological data, viral hepatitis, alcoholic liver disease, and non-alcoholic fatty liver disease (NAFLD) are the most common etiologies of chronic liver disease (CLD). Autoimmune hepatitis (AIH), primary sclerosing cholangitis (PSC), and primary biliary cirrhosis (PBC) are relatively rare [4]. If left untreated, all types of liver diseases may progress to irreversible liver cirrhosis, liver failure, or even hepatocellular carcinoma. Despite the availability of multiple treatment methods for liver disease, liver transplantation is the treatment of choice for liver cirrhosis or failure. Although a subset of patients have benefited from liver transplantation, <10% of global transplantation needs are met because of a liver donor shortage, high surgical costs, and serious postoperative risks [1,5].

New liver-disease treatments are urgently needed. Mesenchymal stromal cell (MSC)-based therapies are used from bench to bedside for numerous diseases [6]. A meta-analysis showed that MSC administration improves the liver and coagulation functions of patients with end-stage liver disease [7]. The role of MSC-derived extracellular vesicles (EV) in tissue repair and regeneration has been studied in several tissues, including the heart, lung, bone, skin, brain, kidney, and liver [8,9]. Meta-analyses of preclinical trials have shown that exosomes can be beneficial for myocardial infarction [10], acute and chronic respiratory diseases [11], acute kidney injury [12], and osteoarthritis [13]. Li et al. (2013). [14] reported that MSC-EV alleviated liver fibrosis in a preclinical drug-induced liver injury model. Furthermore, MSC-EV alleviated the immune response by reducing proinflammatory cytokines [15]. A growing number of studies are appearing about the therapeutic effects of EV on liver disease, especially liver fibrosis [16]. MSC-EV displayed therapeutic efficacy in several preclinical models of liver fibrosis, acute liver injury (ALI), nonalcoholic steatohepatitis (NASH) or NAFLD, hepatic ischemia-reperfusion injury (I/RI), and AIH.

A systematic and comprehensive understanding of the impact of MSC-EV on liver injury is needed. We performed a meta-analysis of studies using animal models to assess the efficacy of MSC-EV for various chronic and acute liver diseases in terms of liver function recovery, reduction of liver tissue damage, and inhibition of inflammation. We also compared the therapeutic efficacies of MSC-EV and MSC. Subgroup analyses explored the sources of EV and the impact of the injection route on efficacy, which may improve MSC-EV treatment.

## 2. Methods

### 2.1. Search Strategy and Study Selection

We systematically searched the PubMed, EMBASE, Web of Science, and Cochrane Library databases up to February 2022. The search strategy involved a combination of relevant terms, including (“extracellular vesicle” or “EV” or “microvesicle” or “MVs” or “exovesicle” or “exosome” or “microparticle”), (“mesenchymal stromal cells” or “mesenchymal stem cells” or “MSC”), and (“liver disease” or “liver cirrhotic” or “liver fibrosis” or “liver injure” or “liver failure”). Two researchers independently screened the titles and abstracts of the retrieved studies. The search strategy is detailed in the supplementary material, search strategy.

### 2.2. Selection Criteria

Full articles were retrieved based on the criteria described below. The inclusion criteria were as follows: any animal model of liver disease or injury, including drug-induced acute or chronic injury, NASH or NAFLD, I/RI, and AIH; any MSC-EV tissue sources; inclusion of a control group; and information on one of the following outcomes: serum liver function [albumin (ALB), alanine aminotransferase (ALT), aspartate aminotransferase (AST), alkaline phosphatase (ALP), γ-glutamyl transferase (γ-GT)], serum inflammatory cytokines [tumor necrosis factor-α (TNF-α), interleukin-1β (IL-1β), IL-6, interferon-γ (IFN-γ), IL-10], or tissue-related indices [ratio of liver weight to body weight (LW/BW), damaged area (%), and Ishak’s score].

The exclusion criteria were as follows: use of immunodeficient animals; cancer or tumor animal models; EV pretreated, cotreated, or modified (e.g., gene transfection); treatment with conditioned medium (CM) instead of EV; EV not derived from MSC; insufficient information or non-quantitative data; and duplicate study, review, conference abstract, expert opinion, or editorial.

### 2.3. Data Extraction

For each study, two researchers independently extracted relevant data to ensure accuracy. A third researcher resolved any disagreement over study selection and data extraction. When a study reported the same outcome at multiple time points or doses, we selected the intermediate time point or dose. When data were not available in the text, we used GetData Graph Digitizer version 2.25.0.32 software to extract data from graphics.

The following data were extracted from the included studies: study information (first author, publication year, country), animals (species, sex, number, and liver disease model), EV (type and diameter of MSC, delivery route, dose, and number of treatments), and outcome measures.

### 2.4. Quality Assessment

The quality assessment criteria were modified from the CAMRADES [17] checklist, which has 10 parameters: 1. calculation of sample size; 2. animals were randomly allocated; 3. blinded model; 4. blinded outcome assessment; 5. appropriate animal model; 6. use of anesthetics without significant protective or toxic effects on the liver; 7. temperature control; 8. statement of compliance with animal welfare regulations; 9. peer-reviewed journal; and 10. statement of potential conflict of interests. Reporting quality was evaluated by two independent researchers. Disagreements were resolved by discussion.

### 2.5. Statistical Analysis

Statistical analysis was performed using RevMan version 5.4.1, and the results are presented as forest plots. *P*-values < 0.05 were considered indicative of statistical significance. Continuous outcomes are expressed as standardized mean differences (SMD) with 95% confidence intervals (CIs). Heterogeneity was analyzed using the I^2^ statistic. I^2^ values < 50% were considered indicative of low or moderate heterogeneity, and a fixed-effects model was used for the meta-analysis. I^2^ ≥ 50% represented significant heterogeneity. A random-effects model was applied, and a funnel plot was generated to check for publication bias.

## 3. Results

### 3.1. Study Selection

**Figure 1:**
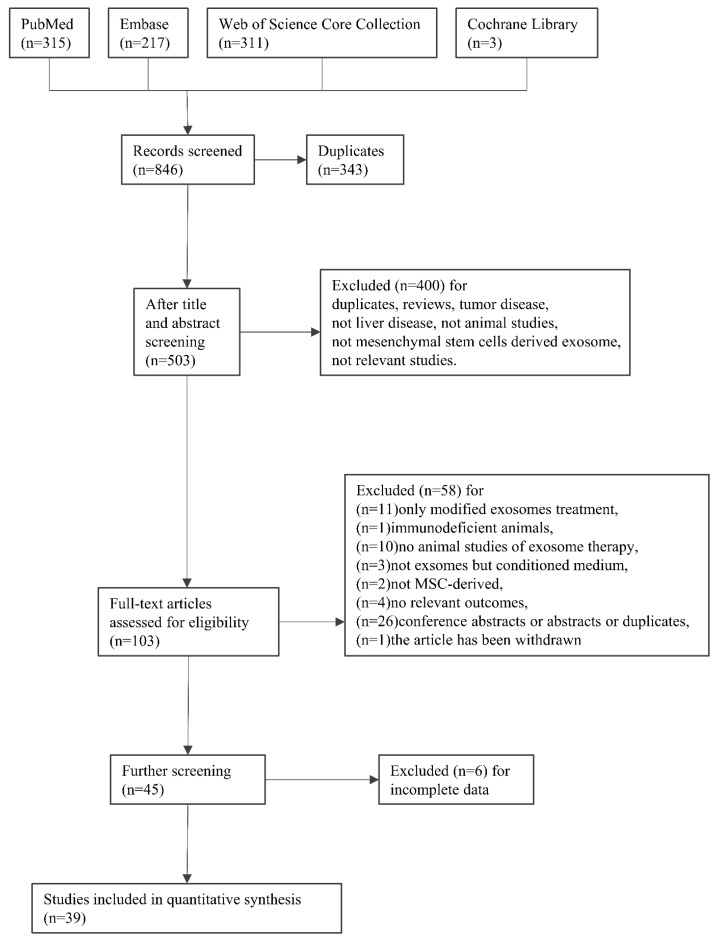
Flow diagram of study selection.

The systematic search yielded a total of 846 records. After removing 343 duplicates, the titles and abstracts of 503 studies were screened, and 104 were retained for full-text evaluation. Ultimately, 40 studies were included in the quantitative synthesis. A flowchart of the study selection process is shown in Figure 1.

### 3.2. Characteristics of the Eligible Studies

In total, 39 studies [15,16,18,19,20,21,22,23,24,25,26,27,28,29,30,31,32,33,34,35,36,37,38,39,40,41,42,43,44,45,46,47,48,49,50,51,52,53,54] were included, containing control and EV groups, among which 11 [15,23,26,27,29,32,42,45,46,50,54] added MSC group. The control group received phosphate-buffered saline, saline, or a vehicle. Table 1 lists the main characteristics of the eligible studies.

The sources of MSCs were humans (n = 25) [14,18,19,20,21,22,23,24,25,26,29,30,32,34,37,38,39,40,41,43,44,46,50,52,53,54], mice (n = 10) [15,31,36,37,38,42,47,48,49,51], and rats (n = 5) [27,28,33,35,45]. MSCs originated from multiple tissues, including bone marrow stromal cells (BMSCs) (n = 16) [15,19,26,27,28,31,33,35,37,42,45,47,48,49,50,51], umbilical cord-derived mesenchymal stem cells (UCMSCs) (N = 8) [14,38,41,43,44,52,53,54], adipose tissue-derived stem cells (ADSCs) (n = 7) [20,21,22,32,36,39,46], embryonic stem cells (ESCs) (n = 4) [23,24,30,40], amnion stem cells (AMSCs) (N = 2) [18,25], menstrual blood-derived stem cells (MenSC) (N = 1) [34], and tonsil-derived stem cells (TSCs) (n = 1) [22]. Gupta et al. [20] and Haga et al. [37] applied two types of MSC-derived EV to establish liver damage models.

The most advanced technology used for EV isolation was ultracentrifugation. EV were characterized in terms of their size distribution, abundance, and surface marker expression (CD9/CD63/CD81). The most common route of EV administration was intravenous (i.v.) injection (n = 31), especially into the tail vein, followed by intraperitoneal (i.p.) [19,24,37,47], intrasplenic (i.s.) [23,40], femoral artery [42], penile vein [25], caudal vein [44], inferior cava [50], hepatic portal vein [35], direct liver injection [14], and oral gavage [41]. One study did not mention the route of EV delivery [53]. The dose of EV varied greatly, including in terms of the absolute protein amount, particle number, and dose by body weight. Most studies used single-dose treatment after damage, although 14 injected multiple doses, and 3 applied the treatment before damage was inflicted [34,50,51].

### 3.3. Meta-Analyses of MSC-EV vs. Placebo Control

#### 3.3.1. MSC-EV Therapy Improves Liver Function

Serum liver function was reported in 35 studies, in all of which MSC-EV therapy significantly restored liver function in models of acute and chronic liver disease.

**Figure 2:**
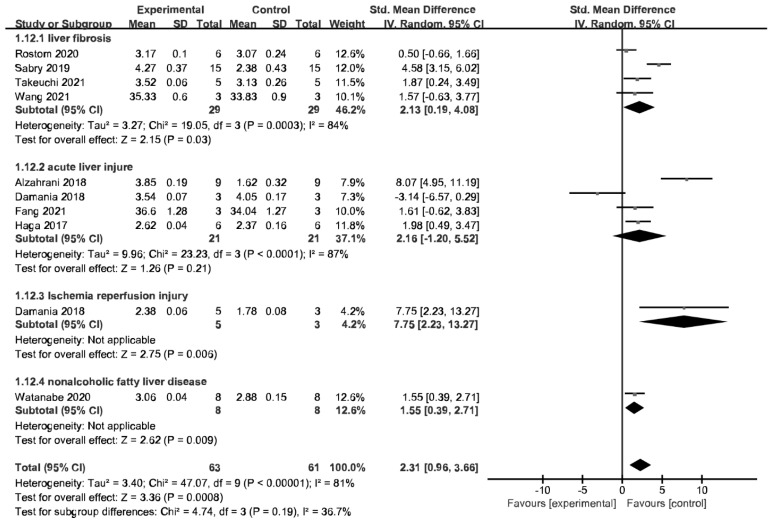
Forest plot of the efficacy of MSC-EV therapy on liver function, albumin (ALB).

Nine studies reported serum ALB levels (nine comparisons). The pooled analysis showed that the serum ALB level of the MSC-EV group was significantly higher than that of the placebo control group (Figure 2). Subgroup analysis showed that MSC-EV therapy significantly increased ALB in liver fibrosis, IRI, and NAFLD but not in ALI (*P* = 0.21) (Figure 2).

**Figure 3:**
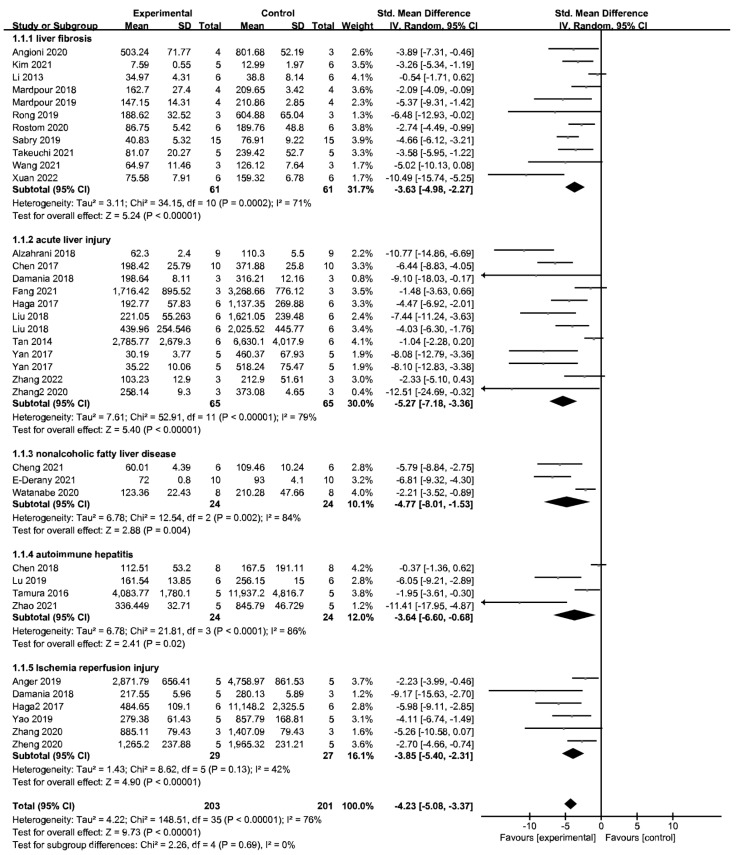
Forest plot of the efficacy of MSC-EV therapy on liver function, alanine aminotransferase (ALT).

Serum ALT was evaluated in 35 studies (36 comparisons) and serum AST in 28 studies (30 comparisons). The pooled analysis showed that MSC-EV lowered the ALT level compared with the placebo control (Figure 3), as well as the AST level (Figure S1). Subgroup analysis showed that the improvement of MSC-EV on liver function was more obvious in chronic liver diseases.

Nine studies reported serum ALP level and four studies reported serum γ-GT level. The pooled analysis illustrated that MSC-EV reduced the ALP (Figure S2) and γ-GT levels (Figure S3) compared with the placebo control. Subgroup analysis showed that the MSC-EV group’s serum ALP level was lower than the control in liver fibrosis, ALI, and IRI (Figure S2). Similarly, the MSC-EV group’s serum γ-GT level was significantly lower in liver fibrosis and ALI, although this was based on a small number of studies (Figure S3).

#### 3.3.2. MSC-EV Therapy Achieve Histological Improvement

Analysis of liver tissue was conducted in 20 studies. Eight studies (nine comparisons) reported LW/BW, which was used as a liver index (five for liver fibrosis, two for ALI, and two for NAFLD). The pooled analysis indicated no significant difference in liver index between the two groups (Figure S4).

**Figure 4:**
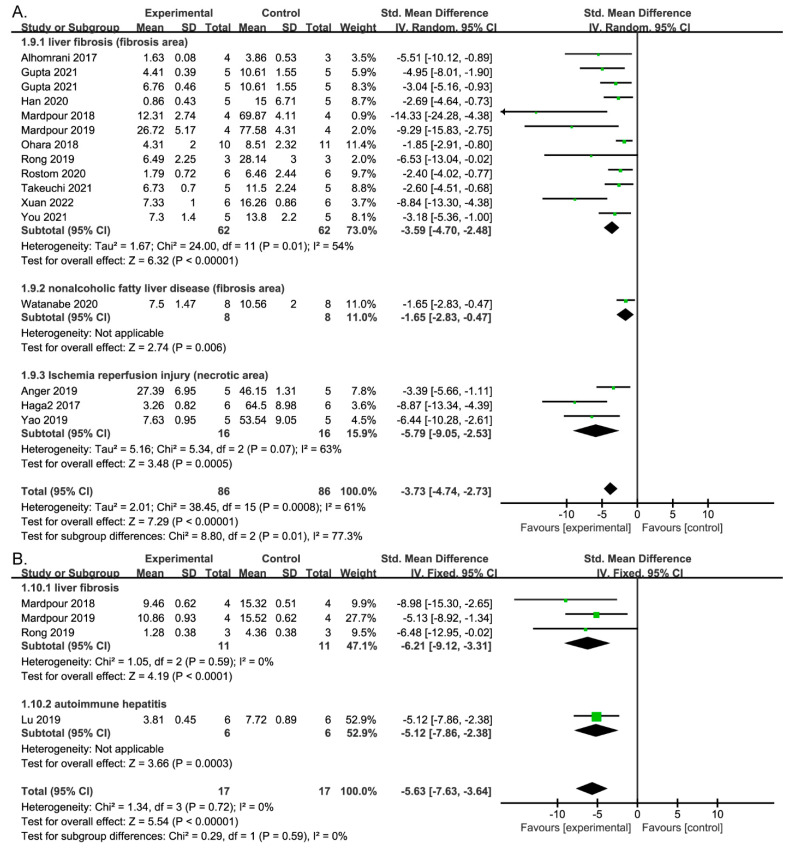
Forest plot of the efficacy of MSC-EV therapy on histological analysis. (**A**) Forest plot of the percentage of damage area; (**B**) Forest plot of Ishak’s score.

A histopathological examination was performed to quantify the extent of liver damage. Twelve studies of liver fibrosis and one of NAFLD reported the fibrotic area (%), as determined by Masson’s trichrome or Sirius red staining. The necrotic area (%), determined by hematoxylin and eosin (H&E) staining, was evaluated in three studies of I/RI. The fibrotic and necrotic areas in the liver were significantly smaller with MSC-EV treatment compared with placebo (Figure 4A). Subgroup analysis showed no difference in effect size among liver fibrosis, NAFLD, and I/RI. This result is in line with those for the Ishak score, which provides a measure of the degree of hepatic inflammatory necrosis and fibrosis. A pooled analysis of four studies showed that MSC-EV reduced the Ishak score (Figure 4B).

#### 3.3.3. MSC-EV Therapy Mitigates Excessive Inflammatory Response

Twelve studies (thirteen comparisons: two in liver fibrosis, six in ALI, two in IRI, and three in AIH) evaluated serum inflammatory cytokines. The pooled analysis showed that MSC-EV significantly reduced the serum levels of TNF-α, IL-1β, IL-6, IFN-γ, but significantly increased the serum level of IL-10 compared to placebo. (Table 2).

#### 3.3.4. Further Subgroup Analyses 

ALT and AST levels were analyzed to explore the therapeutic effect according to MSC sources, EV administration route and frequency, and animal species (Supplementary Tables S1 and S2).

In the analysis of liver fibrosis, no difference in efficacy (based on the ALT or AST level) was observed among animal species. In addition, the two indicators displayed inconsistent results. Multiple treatments may be superior to single treatments in terms of the ALT level, but no difference was seen for the AST level. While i.v. injection was preferable to i.p. injection in terms of the AST level, there was no difference in the ALT level among i.v., i.p., and i.s. injections. Regarding ALT, there was no significant difference between MSC-EV and placebo control, except for UCMSC-derived EV (*P* = 0.36), and the treatment outcomes were similar among EV derived from BMSCs, AMSCs, and ESCs. EV from BMSCs, UCMSCs, and AMSCs produced similar outcomes in terms of AST.

In the subgroup analysis of ALI, the ALT and AST levels showed similar trends. EV exerted a greater therapeutic effect in rats than mice. No difference in efficacy was observed according to MSC type (BMSC. vs. UCMSC vs. AMSC) or administration route (i.v. vs. i.p. injection). The i.s. administration route for ESCs did not exhibit significantly greater efficacy than the other routes (*P* = 0.09).

In the subgroup analysis of NAFLD, there was no difference in effect size among MSC tissue sources (BMSCs vs. UCMSCs vs. AMSCs). MSC exerted a greater therapeutic effect in rats than mice, and multiple treatments were superior to single ones in terms of the ALT but not the AST level.

In the subgroup analysis of AIH, a single treatment was superior to multiple treatments in terms of the ALT level but not the AST level. Furthermore, i.p. injection showed greater benefit than i.v. injection in terms of the AST but not the ALT level.

In the subgroup analysis of hepatic I/RI, no difference in the effect size for the AST or ALT level was observed between MSC sources (BMSCs vs. UCMSCs) or animal species (mouse vs. rat).

### 3.4. MSC-EV Therapy Displays No Inferior Performance to MSC

Eleven studies [15,23,26,27,29,32,42,45,46,50,54] were included in the analysis of the efficacy of MSC-EV and MSCs in liver models. The pooled analysis demonstrated no significant difference between MSC-EV and MSCs therapy in terms of serum ALB, ALT, AST (*P* = 0.15), ALP or TNF-α. The damage area with MSC-EV was significantly smaller than that with MSC (Table 3).

### 3.5. Sensitivity Analysis and Quality Assessment

The sensitivity analysis showed that no single study affected any of the pooled results significantly. However, the high level of heterogeneity could not be reduced by excluding any single study (Supplementary Tables S3–S9).

The results of the 10-item quality assessment are presented in Table S10. The scores of the studies ranged from 4 to 7 (out of 10). None of the studies detailed the sample size calculation, blinding during the establishment of animal models, or outcome assessments. Thirteen of the studies stated that random allocation was used, and fifteen reported temperature control. All studies used appropriate animal models and were peer-reviewed publications. All animals were given an anesthetic without significant protective or toxic effects on the liver. Compliance with animal welfare regulations (37 of 39 studies) and potential conflict of interests (34 of 39 studies) were described in most studies. The publication bias results are shown in Figure S5.

## 4. Discussion

This systematic review comprehensively analyzed the preclinical efficacy of MSC-EV for liver disease. Pooled estimates from meta-analyses indicated that MSC-EV significantly enhanced liver functions (reflected in ALB, ALT, AST, ALP, and γ-GT levels), repaired injured liver tissue (reflected in the damaged area and Ishak’s score), reduced inflammatory factors (TNF-α, IL-1β, IL-6, IFN-γ), and increased an anti-inflammatory factor (IL-10). In subgroup analyses of liver disease models, MSC-EV exerted therapeutic effects in acute (ALI, I/RI) and chronic (liver fibrosis, NAFLD, AIH) liver diseases. Consequently, our meta-analysis provides important evidence for the entry of EV into clinical trials.

ALB is mainly synthesized in the liver, reflecting the reserve function of the liver. ALB reduction is often suggestive of severe late-stage liver injury [55,56]. ALT and AST are the most commonly measured laboratory serum markers in the clinical diagnosis and treatment of patients with liver disease [55,56]. These are also the most frequent indicators in the studies we included. ALT and AST are mainly distributed in hepatocytes. If the liver is damaged, ALT and AST in the liver cells enter the blood, causing elevated levels of ALT and AST in the blood [55,56]. The increased activity of ALP and γ-GT mainly reflects cholestasis caused by intrahepatic or extrahepatic biliary obstruction [57]. We found an increase in ALB and a decrease in ALT, AST, ALP, and γ-GT levels after MSC-EV treatment, suggesting that MSC-EV has a protective effect on liver repair.

Liver biopsy is considered the gold standard for evaluating the grade of liver injury and staging of liver fibrosis in patients with hepatitis [58]. Various scoring systems, such as the Batts-ludwig, METAVIR, and Ishak scoring systems, have been developed to stage and grade histological samples obtained from the liver. These scoring systems can be used regardless of the cause of the disease [58,59,60]. In our study, the Ishak score system is the most mentioned. The Ishak score classifies fibrosis into seven categories (0–6 points). Stages 1 and 2 represent mild fibrosis with no bridging. Stages 4 and 5 represent late bridging fibrosis and nodule formation [59,60]. We observed a reduction in the Ishak score of the liver tissue as well as in the area of fibrosis in the MSC-EV treatment group. MSC-EV promotes the proliferation of liver cells, reverses liver fibrosis, delays the progress of chronic hepatitis, and may reduce the incidence of late liver disease.

Inflammation is an immune state that underlies the body’s natural physiology under healthy conditions [61]. Inflammation must be carefully regulated to maintain dynamic equilibrium. Failure to control inflammation can lead to multiple organ tissue damage or loss [61,62]. Regulating inflammation is an integral part of liver health. The development of chronic liver disease is associated with persistent inflammation that eventually leads to fibrosis and cirrhosis [62,63,64]. Cytokines are low molecular weight glycoproteins that induce local inflammation and acute systemic reactions. Cytokines include TNF, IL-6 and the interferon (α, β and γ) family. TNF-α, Il-1β, IL-6, and IFN-γ are considered Proinflammatory cytokine, while IL-4, IL-10, and IL-1 receptor antagonist are considered anti-inflammatory cytokines [63,64]. These inflammatory mediators are associated with disease severity and organ failure in the liver []. We noticed the expression of serum inflammatory cytokines (TNF-α, IL-1β, IL-6 and IFN-γ) in the MSC-EV group was significantly lower than that in the PBS group. A study also noted decreased expression of inflammatory factors in liver tissue [33]. It indicates that MSC-EV plays a role in controlling systemic inflammatory factors and the liver inflammatory response.

Our systematic analysis showed that the sources of MSC-EV were diverse, as were the experimental methods, intervention characteristics, dosing schemes, and study designs. To identify factors related to MSC-EV efficacy, we performed subgroup analyses according to MSCs type, EV administration frequency and route, and animal species. In terms of serum ALT and AST levels, the different sources of MSCs (BMSCs, USMSCs, or ADSCs), routes of administration (i.v. or i.p.), and numbers of treatments (single or multiple) were all efficacious. However, more evidence of the therapeutic efficacy of i.s. injection of ESC-EV is needed. Multiple infusions were considered to have a more lasting effect [65,66]. In contrast, this meta-analysis shows that multiple injections did not exert a greater benefit. It is no coincidence. A recent meta-analysis of MSC-EV therapy for osteoarthritis showed that once-weekly administration was better than multiple administrations [13]. In addition, a meta-analysis of MSC for liver disease suggested that single-dose administration exerted a greater therapeutic effect than multiple doses [7]. Still, it’s worth noting that the effect of EV is dose-dependent [41,46]. 

MSC for liver disease has been studied in great depth and has entered clinical trials [67,68]. Studies have shown that MSC can improve liver function, reverse liver fibrosis, relieve clinical symptoms, and reduce mortality [69,70,71,72]. We compared the therapeutic effects of MSC with those of MSC-EV and found EV treatment outcomes did not differ significantly from those of MSC in terms of serum liver function and inflammation, even being superior in terms of histopathology. This is consistent with the findings of a meta-analysis of stem cell-derived EV therapy for acute kidney injury [12]. Therefore, the therapeutic effect of EV is comparable to that of MSC. The potential safety risks of cell injection to induce immune responses and tumorigenic potential must be taken in to account [73]. Compared to MSC, EV are free from the risk of cellular immunological rejection, easier to generate in advance, more stable (which is beneficial for storage and transport), and more amenable to control in terms of quality and quantity [74,75,76]. Therefore, MSC-EV may be a safer cell-free alternative than whole-cell injection. However, few studies have addressed the adverse effects of EV treatment.

MSC-EV has been shown to be beneficial in small animal models for a range of acute and chronic liver diseases. However, the efficacy of EV for liver disease needs to be supported by large animal studies. In addition, uniform and standardized guidelines on EV production and intervention methods are needed before their clinical application.

Several limitations to this study should be considered. First, although we demonstrated that injection of stem cell-derived EV improves liver injury, we did not identify the underlying mechanisms responsible. Second, although we used a random-effects model and performed a sensitivity analysis, significant heterogeneity between studies was detected, which may have reduced the reliability of the results. Third, the number of studies of individual diseases was insufficient, especially NAFLD and AIH. However, few studies have analyzed the levels of inflammatory cytokines. Moreover, we did not consider the impact of EV size, separation technique, or injection dose, which may explain the heterogeneity. In preclinical studies, the quality of reporting and potential risk of bias in the study design are unclear. Lastly, most data were extracted from graphics, and so may not fully reflect the real data.

## 5. Conclusions

MSC-EV can improve liver function and inflammation, reverse liver fibrosis, and promote liver tissue healing in preclinical liver disease models. MSC-EV is expected to be a safe and effective cell-free therapy. Before EV enters clinical research, it is necessary to carry out large-scale animal studies with strict design on the therapeutic effect of EV.

## Figures and Tables

**Table 1:** Character of included studies.

Study (Year) Country	Species Sex	Injure Model	Cell Source of EV	Isolation Technique	Diameter (nm)	EV Treatment Group (Method/Dose)	Number	Therapy Time	Measurement Time	Available Outcomes
Alhomrani (2017) Australia [18]	C57BL/6 mice male	CCL_4_ induce liver fibrosis	hAMSC	ultracentrifugation	40–100	i.v./1 µg (~24 × 10^6^ particles, 350 µL)	C:6 T:6	three doses weekly for 4 weeks after injure	12 weeks after treatment	fibrosis area% (sirius red staining)
Alzahrani (2018) China [33]	albino rats male	Diethylnitrosamine induce ALI	Rat-BMSC	ultracentrifugation	30–100	i.v./250 µg	C:9 T:9	single after injure	4 weeks after therapy	serum ALT, AST, ALB, ALP
Anger (2019) Germany [50]	C57BL/6 mice female	I/RI	hBMSC	ultracentrifugation	160 ± 57	inferior cava/1 × 10^9^ particles	C:5 T:5	single before injure	24 h/48 h/72 h after reperfusion	serum ALT, AST; necrosis area% (hematoxylin and eosin staining).H&E
Angioni (2020) Italy [19]	FVB.129P2-Abcb4tm1Bor mice male	PSC	hBMSC	Ultrafiltration	45–372	intraperitoneal/9.1 × 10^9^ particles/mL (100 µL)	C:3 T:4	once a week for 3 weeks after injure	3 weeks after treatment	Serum of ALT, ALP
Chen (2017) China [34]	C57BL/6 mice male	D-GalN/LPS induce FHF	MenSC	commercial kits	30–100	tail vein/1 μg/μL	C:10 T:10	single before injure	after therapy	serum ALT, AST; serum IL-6, IL-1β, TNF-α
Chen (2018) China [47]	C57BL/6 mice male	AIH	mouse-BMSC	ultracentrifugation	30–100	intraperitoneal/20 μg/mL	C:8 T:8	day 21/28/35 after injure	21 days after therapy	serum levels of ALT, AST; serum TNF-α, IL-17, IL-1β
Cheng (2021) China [44]	Sprague-Dawley rats male	NAFLD	hUCMSC	commercial kits	96	caudal vein/100 μg (500 μL)	C:6 T:6	once a week for 2 months	8 weeks after model	serum ALT, AST; liver index
Damania (2018) India [35]	Wistar rats male	IRI and CCL_4_ induce acute liver injury	rat-BMSC	differential centrifugation	165 ± 3	hepatic portal vein/50 μg	C:3 T:5 C:3 T:3	single after injure	24 h/48 h/72 h after therapy	serum ALT, AST, ALB, TBIL
E-Derany (2021) Egypt [45]	Sprague-Dawley rats NA	NAFLD	rat-BMSC	differential centrifugation	NA	tail vein/(15 µg/kg), (30 µg/kg) or (120 µg/kg)	C:10 T:10	twice weekly for 6 weeks after injure	12 weeks after model	serum AST, ALT
Fang (2021) China [36]	Mice male	CCL_4_ induce ALI	mouse-ADSC	commercial kits	NA	tail vein/200 mL	C:3 T:3	single 10 h after CCl4	3 day after treatment	LW/BW; serum AST, ALT, ALB, γ-GT; The histopathological score(HE staining )
Gupta (2021) India [20]	Swiss albino mice female	CCL_4_ induce liver fibrosis	hADSC/WJMSC	commercial kits	40–120	tail vein/250 μg	C:5 T:5	single after injure	day7 after treatment	liver index; Histopathological analysis of Masson’s Trichrome staining and Sirius Red Staining Red area
Haga (2017) USA [37]	C57Bl/6 mice male	TNF-α/D-GalN induce ALF	hBMSC/mouse-BMSC	ultracentrifugation	116 ± 46	intraperitoneal/2 × 10^10^ particles/body	C:6 T:6	single after injure	6 h after therapy	Serum ALT, AST, ALP
Haga2 (2017) USA [51]	C57BL/6 mice male	I/RI	mouse-BMSC	ultracentrifugation	115 ± 48	tail vein/2 × 10^10^ particles	C:6 T:6	single before injure	6 h after reperfusion	serum ALT, AST, ALP, TBIL; necrosis area% (H&E) staining
Han (2020) Korea [21]	C57BL/6 mice male	TAA induce liver fibrosis	hADSC	tangential flow filtration(TFF)	94.2 ± 4.7	tail vein/200 μL (1 × 10^7^; 1 × 10^8^)	C:5 T:5	single after injure	24 h after treatment	LW/BW; collagen areas (Masson’s trichrome); Hyp level in tissue
Jiang (2019) China [38]	C57BL/6 mice male	LPS/D-GalN-induce ALF	hUCMSC	centrifugation	100	tail vein/100 μg (250 μL)	C:6 T:6	single 1 h after injure	12 h after therapy	ALT, AST; serum levels of IL-6, IL-1β
Kim (2021) Korea [22]	C57BL/6 mice Male	CCL_4_ induce liver fibrosis	hTMSC	differential centrifugation	50–290	i.v./150 mg (100 μg/mL)	C:5 T:6	once a week for 3 weeks after injure	48 h after last treatment	LW/BW; serum ALT, AST
Li (2013) China [14]	mice	CCL4 induce liver fibrosis	hUCMSC	ultracentrifugation	40–100	liver directly injected/250 μg (330 μL)	C:6 T:6	single after injure	3 weeks after therapy	serum AST, ALT
Liu (2018) China [39]	C57BL/6J mice	LPS/D-GalN or TNF-α/D-GalN induce ALF	mouse-ADSC	commercial kits	40–100	tail vein/400 μg (300 μL)	C:6 T:6	single after injure	6 h after treapy	Serum ALT, AST; serum TNF-α, IFN-γ, IL-1β, IL-6, IL-18
Lu (2019) China [48]	C57BL/6 mice male	AIH	mouce-BMSC	ultracentrifugation	40–100	tail vein/2 µg/g (200 µL)	C:6 T:6	Day 21/35 after injure	3 weeks after therapy	serum ALT, AST; The histological scoring Ishak; serum IL-1β, IL-6, IL-17, and IL-10
Mardpour (2018) Iran [23]	wistar rats male	TAA induce liver fibrosis	hES-MSC	ultracentrifugation	NA	infused intrasplenicly/350 µg (4 × 10^6^ cells, 400 µL)	C:4 T:4	single after injure	4 weeks after therapy	serum ALT, ALP, and GGT (γ-GT); IL-10 and TNF-α in serum; Ishak’s score; fibrous-positive area (masson-trichrome);
Mardpour (2019) Iran [24]	wistar rats male	TAA induce liver fibrosis	hES-MSC	ultracentrifugation	190.8 ± 18	intraperitoneal/350 μg (400 μL)	C:4 T:4	single after injure	4 weeks after therapy	serum ALT, AST, ALP, GGT; serum TNF-α, IL10; Ishak’s score; the positive fibrosis area% (Masson trichrome)
Ohara (2018) Japan [25]	Sprague-Dawley rats male	CCL_4_ induce liver fibrosis	hAMSC	Ultracentrifugation	80–110	penile vein/15 μg/kg and 20 μg/kg (200 μL)	C:15 T:15	single after injure	4 weeks after therapy	fibrosis area (Masson trichrome staining)
Rong (2019) China [26]	Sprague-Dawley rats female	CCL_4_ induce liver fibrosis	hBMSC	ultracentrifugation	30–100	tail vein/250 mg (500 μL)	C:3 T:3	single after injure	4 weeks after treatment	serum ALT, AST, ALP, γ-GT; liver index%; Hyp in liver tissue; Ishak scoring; Collagen area% (Masson, and Sirius red)
Rostom (2020) Egypt [27]	Sprague-Dawley albino rats male	CCL_4_ induce liver fibrosis	rat-BMSC	ultracentrifugation	113.7	tail vein/80 μg	C:6 T:6	single after injure	4 weeks after treatment	serum AST, ALT, ALB; Ishak grade; area percentage of collagen fibers
Sabry (2019) Egypt [28]	white albino rats female	CCL_4_ induce liver fibrosis	rat-BMSC	ultracentrifugation	NA	tail vein/4 μg (1 mL)	C:15 T:15	twice weekly for 4 weeks after injure	4 weeks after treatment	serum ALT, ALB
Takeuchi (2021) Japan [29]	C57BL/6 mice male	CCL4 induce liver fibrosis	hADSC	ultracentrifugation	NA	i.v./2 μg, or 5 μg	C:5 T:5	single after injure	4 weeks after treatment	serum ALT, ALP, ALB; Sirius Red-stained areas; Hyp levels
Tamura (2016) Japan [15]	C57B6 mice male	con-A induce AIH	mouse-BMSC	ultracentrifugation	135	i.v./10 μg (0.1 mL)	C:5 T:5	once and three times after injure	after therapy	plasma ALT; necrotic area (hematoxylin-eosin staining)
Tan (2014) Singapore [40]	C57BL/6 mice male	CCl_4_ induce ALI	hESC	tangential flow filtration (TFF)	55–65	intrasplenic injection (i.s.)./0.4 μg (100 μL)	C:6 T:6	24 h after injure	24 h after therapy	serum AST, ALT
Wang (2021) China [30]	ICR mice Male	CCL_4_ induce liver fibrosis	hESC	NA	120–140	i.v./NA	C:3 T:3	twice a week for 4 weeks after injure	1/2/3/4 week after treatment	serum ALT, AST, ALB
Watanabe (2020) Japan [46]	Mc4r-KO C57BL/6J mice NA	NAFLD	hADSC	ultracentrifugation	NA	tail vein/1.0 mg, 2.5 mg, or 5.0 mg	C:8 T:8	single after injure	24 h after therapy	LW/BW; serum ALT, ALP, ALB
Xuan (2022) China [31]	C57BL/6 J mice Either sex	TAA induce liver fibrosis	mouse-BMSC	centrifugation and filter	NA	tail vein/5 × 10^5^ cells	C:12 T:12	single after injure	day 21 after treatment	serum ALT, AST level; positive staining areas (Sirius Red staining)
Yan (2017) China [41]	BALB/c-nu/nu mice female	CCl_4_ induce ALF	hUCMSC	ultracentrifugation	NA	tail vein and oral gavage/8 mg/kg, 16 mg/kg, 32 mg/kg; the final 20 mg/mL	C:20 T:20	single after injure	72 h after therapy	Serum AST, ALT; Serum IFN-α, IL-1 α, IL-6, TNF-α
Yao (2019) China [52]	Sprague-Dawley rats male	IRI	hUCMSC	ultracentrifugation	178 ± 64	tail vein/10 mg/kg	C:5 T:5	single after injure	after 24 h reperfusion	serum ALT, AST, ALP; necrosis area%(H&E); serum IL-1b, IL-6, and TNF-a
You (2021) Korea [32]	C57BL/6 mice male	TAA induce liver fibrosis	ADSC	tangential flow filtration (TFF)	117 ± 7	i.v./(1 × 10^7^ particles), or (1 × 10^8^ particles)	C:5 T:5	single or three times after injure	24 h after treatment	Serum AST; fibrotic areas (Sirius Red)
Zhang (2022) China [42]	C57BL/6 mice Male	THS induce ALI	mouse-BMSC	gradient centrifugation	90–142	femoral artery/20 μg	C:5 T:5	single after resuscitation	72 h after resuscitation	serum ALT, AST, LDH
Zhang1 (2020) China [53]	Sprague Dawley rats male	IRI	hUCMSC	ultracentrifugation	NA	NA	C:3 T:3	NA	24 h after reperfusion	serum ALT, AST;
Zhang2 (2020) China [43]	C57BL/6 mice male	LPS + D-GalN induce ALF	hUCMSC	ultracentrifugation	30–150	tail vein/100 mg	C:3 T:3	1 h after injure	12 h after treatment	Serum ALT; LW/BW; IL-6, IL-1β, IL-18 in serum
Zhao (2021) China [49]	BALB/c mice	con-A induce AIH	mouse-BMSC	ultracentrifugation	120	i.v./5 mg/kg (100 µL)	C:5 T:5	single after injure	8 h after treatment	serum ALT, AST level; inflammatory cytokine levels; TNF-α, INF-γ, IL-1β, IL-6, IL-12
Zheng (2020) China [54]	C57BL/6 mice male	IRI	hUCMSC	ultracentrifugation	30–150	i.v./100 µg/100 µL	C:5 T:5	single after injure	6 h after reperfusion	sreum ALT, AST, LDH; IFN-γ, IL-6, and TNF-α in serum;

EV: extracellular vesicle; MSC: mesenchymal stem cell; NA: not available; C: placebo control group; T: MSC-EV treatment group; ALI: acute liver injure; NAFLD: nonalcoholic fatty liver disease; AIH: autoimmune hepatitis; IRI: ischemia-reperfusion injury; PSC: primary biliary cirrhosis; BMSC: bone marrow mesenchymal stem cell; UCMSC: umbilical cord mesenchymal stem cell; ADSC: adipose-derived mesenchymal stem cell; ESC: embryonic stem cell; AMSC: amnion-derived mesenchymal stromal cell; TSC: tonsil-derived mesenchymal stromal cell; MenSC: menstrual blood-derived mesenchymal stem cell; i.v.: intravenous injection; CCl_4_:carbon tetrachloride; TAA; thioacetamide; D-GalN/LPS; D-galactosamine (D-GalN) and lipopolysaccharide (LPS); DEN: diethylnitrosamine; ALB: albumin; ALT: alanine aminotransferase; AST: aspartate aminotransferase; ALP: alkaline phosphatase; γ-GT: γ-glutamyl transferase; TNF-α: tumor necrosis factor-α; IL: interleukin; TFN-γ; interferon-γ; LW/BW: ratio of liver weight to body weight.All studies used mouse or rat models and investigated a variety of liver conditions. Most studies used drug-induced chronic, or ALI, models. The drugs mainly involved carbon tetrachloride (CCl_4_), thioacetamide (TAA), D-galactosamine (D-GalN), lipopolysaccharide (LPS) (D-GalN/LPS), TNF-α/D-GalN, and diethylnitrosamine (DEN). Sixteen studies used chronic liver injury or liver fibrosis models [14,18,19,20,21,22,23,24,25,26,27,28,29,30,31,32]. Only one study used an FVB (Abcb4^tm1Bor^) mouse model of PSC, which was classified as a liver fibrosis model. Eleven studies established the ALI or ALF models [33,34,35,36,37,38,39,40,41,42,43], in which a traumatic hemorrhagic shock (THS) model was also used [42]. Three studies established NAFLD models based on a high-fat diet [44,45,46]. Four studies established AIH models [15,47,48,49]; two used hepatic injection of S100 [47,48], and two used Con-A [15,49]. Six studies established hepatic ischemia reperfusion injury (I/RI) models [35,50,51,52,53,54] by clamping the hepatic artery and portal vein. Two studies established two damage models: one established two ALF models (induced by LPS/D-GalN and TNF-α/D-GalN) [39] and the other established hepatic I/RI and CCl4-induced ALI models [35].

**Table 2:** The efficacy of MSC-EV therapy on inflammatory cytokine.

Outcomes	Number of Animals	Std. Mean Difference (95%CI)	Test for Effect (*P* Value)	Heterogeneity, I^2^
TNF-α	104	−4.60 [−6.45, −2.75]	*P* < 0.01	I^2^ = 79%
IL-1β	110	−4.34 [−6.02, −2.66];	*P* < 0.01;	I^2^ = 77%
IL-6	102	−5.26 [−7.07, −3.45];	*P* < 0.01	I^2^ = 70%
IFN-γ	32	−2.94 [−4.11, −1.78];	*P* < 0.01	I^2^ = 0%
IL-10	28	3.66 [2.14, 5.17]	*P* < 0.01	I^2^ = 0%

Abbreviations: TNF-α: tumor necrosis factor-α; IL: interleukin; TFN-γ: interferon-γ.

**Table 3:** Comparing the efficacy of MSC-EV therapy with MSCs therapy.

Outcomes	Number of Animals	Std. Mean Difference (95%CI)	Test for Effect (*P* Value)	Heterogeneity, I^2^
ALB	38	−0.73 [−2.97, 1.51]	*P* = 0.52	I^2^ = 86%
ALT	112	−0.39 [−1.34, 0.55]	*P* = 0.41;	I^2^ = 78%
AST	68	−0.88 [−2.08, 0.31]	*P* = 0.15;	I^2^ = 76%
ALP	34	−1.17 [−2.54, 0.19]	*P* = 0.09;	I^2^ = 60%
TNF-α	28	0.01 [−1.11, 1.14]	*P* = 0.98;	I^2^ = 50
the damage area	92	−1.32 [−2.25, −0.38]	*P* < 0.01	I^2^ = 68%

Abbreviations: ALB: albumin; ALT: alanine aminotransferase; AST: aspartate aminotransferase; ALP: al kaline phosphatase; TNF-α: tumor necrosis factor-α.

## Data Availability

All data generated or analyzed during this study are included in this article.

## Supplementary Materials

The following supporting information can be downloaded at: https://www.mdpi.com/article/10.3390/jpm13030441/s1, Table S1: Subgroup analyses of MSC-EV vs. Placebo on ALT result. Table S2: Subgroup analyses of MSC-EV vs. placebo on the AST result. Table S3: Results of sensitivity analyses of the effect of MSC-EV therapy vs. placebo on ALB level. (Random-effects model). Table S4: Results of sensitivity analyses of the effect of MSC-EV therapy vs. placebo on ALT level. (Random-effects model). Table S5: Results of sensitivity analyses of the effect of MSC-EV therapy vs. placebo on AST level. (Random-effects model). Table S6: Results of sensitivity analyses of the effect of MSC-EV therapy vs. placebo on the damaged area. (Random-effects model). Table S7: Results of sensitivity analyses of the effect of MSC-EV therapy vs. placebo on TNF-α (Random-effects model). Table S8: Results of sensitivity analyses of the effect of MSC-EV therapy vs. placebo on IL-1β. (Random-effects model). Table S9: Results of sensitivity analyses of the effect of MSC-EV therapy vs. placebo on IL-6. (Random-effects model). Table S10: CAMARADEA quality assessment. Figure S1: Forest plot of the efficacy of MSC-EV therapy on aspartate aminotransferase (AST). Figure S2: Forest plot of the efficacy of MSC-EV therapy on alkaline phosphatase (ALP). Figure S3: Forest plot of the efficacy of MSC-EV therapy on γ-glutamyl transferase (γ-GT). Figure S4: Forest plot of the efficacy of MSC-EV therapy on the liver index. Figure S5: Funnel pots for ALB, ALT, AST, ALP, and damage area on MSC-EVs vs Placebo (A-E); ALT on MSC-EVs vs MSC (F).

## Abbreviations

EV: extracellular vesicle; MSC: mesenchymal stem cell; ALI: acute liver injury; NAFLD: nonalcoholic fatty liver disease; AIH: autoimmune hepatitis; I/RI: ischemia-reperfusion injury; PSC: primary biliary cirrhosis; BMSC: bone marrow mesenchymal stem cell; UCMSC: umbilical cord mesenchymal stem cell; ADSC: adipose-derived mesenchymal stem cell; ESC: embryonic stem cell; AMSC: amnion-derived mesenchymal stromal cell; TSC: tonsil-derived mesenchymal stromal cell; MenSC: menstrual blood-derived mesenchymal stem cell; i.v.: intravenous injection; CCl4:carbon tetrachloride; TAA; thioacetamide; D-GalN/LPS; D-galactosamine (D-GalN) and lipopolysaccharide (LPS); DEN: diethylnitrosamine; ALB: albumin; ALT: alanine aminotransferase; AST: aspartate aminotransferase; ALP: alkaline phosphatase; γ-GT: γ-glutamyl transferase; TNF-α: tumor necrosis factor-α; IL: interleukin; TFN-γ; interferon-γ; LW/BW: ratio of liver weight to body weight.
