# Supplementary material for: Pooled Analysis of Mesenchymal Stromal Cell-Derived Extracellular Vesicle Therapy for Liver Disease in Preclinical Models
Source: J Pers Med. 2023 Feb 28;13(3):441. doi: 10.3390/jpm13030441 (PMC10056150; doi:10.3390/jpm13030441)
Supplement: Supplementary file 1 [file jpm-13-00441-s001.zip › jpm-2246755-supplementary.pdf]

Pooled Analysis of Mesenchymal Stromal Cell-Derived Extracellular Vesicle Therapy for Liver Disease in Preclinical Models

Xinru Fang [1,2,3], Feiqiong Gao [2], Qigu Yao [2], Haoying Xu [1,2,3], Jiong Yu [2], Hongcui Cao [2,4,5,*] and Shibo Li [1,*]

[1] Department of Infectious Disease, Zhoushan Hospital, Zhejiang University School of Medicine, Zhoushan 316021, China; xinru_f@zju.edu.cn (X.F.); xuhaoying@zju.edu.cn (H.X.)
[2] State Key Laboratory for the Diagnosis and Treatment of Infectious Diseases, Collaborative Innovation Center for Diagnosis and Treatment of Infectious Diseases, The First Affiliated Hospital, Zhejiang University School of Medicine, Hangzhou 310003, China; 12018208@zju.edu.cn (F.G.); yaoqigu@zju.edu.cn (Q.Y.); yujiong@zju.edu.cn (J.Y.)
[3] Department of Laboratory Medicine, The Fourth Affiliated Hospital, International Institutes of Medicine, Zhejiang University School of Medicine, Yiwu 310003, China
[4] Jinan Microecological Biomedicine Shandong Laboratory, Jinan 250117, China
[5] Key Laboratory of Diagnosis and Treatment of Aging and Physic-Chemical Injury Diseases of Zhejiang Province, 79 Qingchun Rd, Hangzhou 310003, China
* Correspondence: hccao@zju.edu.cn (H.C.); lsb0398@126.com (S.L.); Fax: +86-571-87236459 (H.C.)

Search strategy

PubMed 315

#1="vesicle"[Title/Abstract] OR "extracellular vesicle"[Title/Abstract] OR "exovesicle"[Title/Abstract] OR "microvesicle"[Title/Abstract] OR "MVs"[Title/Abstract] OR "EVs"[Title/Abstract] OR "exosome"[Title/Abstract] OR "exosome"[Title/Abstract] OR "microparticle"[Title/Abstract]

#2="mesenchymal stem cells"[Title/Abstract] OR "mesenchymal stromal cells"[Title/Abstract] OR "MSC"[Title/Abstract]

#3= "Liver"[Title/Abstract] OR "Hepatic"[Title/Abstract]

#4="Cirrhosis"[Title/Abstract] OR "Fibrosis"[Title/Abstract] OR "Fibrose"[Title/Abstract] OR "Disease"[Title/Abstract] OR "Failure"[Title/Abstract] OR "Injure"[Title/Abstract]

#5=#3 AND #4

#6=#1 AND #2 AND #5

Web of Science 311

#1=((((((((TS=(vesicle)) OR TS=(extracellular vesicle)) OR TS=(exovesicle)) OR TS=(microvesicle)) OR TS=(MVs)) OR TS=(EV)) OR TS=(exosome)) OR TS=(cell derived microparticle)) OR TS=(Microparticle)

#2=((TS=(mesenchymal stem cells)) OR TS=(Mesenchymal Stromal Cells)) OR TS=(MSC)

#3=(TS=(Liver)) OR TS=(Hepatic)

#4=((((((TS=(Cirrhotic)) OR TS=(Cirrhosis)) OR TS=(Fibrosis)) OR TS=(Fibrose)) OR TS=(Disease)) OR TS=(Failure)) OR TS=(Injure)

#1 AND #2 AND #3 AND #4

Embase 217

#1=vesicle:ab,ti OR 'extracellular vesicle':ab,ti OR exovesicle:ab,ti OR microvesicles:ab,ti OR mvs:ab,ti OR evs:ab,ti OR exosomes:ab,ti OR 'cell derived

microparticle':ab,ti OR microparticle:ab,ti

#2= 'mesenchymal stem cells':ab,ti OR 'mesenchymal stromal cells':ab,ti OR msc:ab,ti OR 'stem cell':ab,ti

#3=liver:ab,ti OR hepatic:ab,ti

#4=cirrhotic:ab,ti OR 'liver cirrhosis':ab,ti OR fibrosis:ab,ti OR fibrose:ab,ti OR disease:ab,ti OR failure:ab,ti OR injure:ab,ti

#5=#3 AND #4

#1 AND #2 AND #5

Cochane Library 3

#1= (MVS):ti,ab,kw OR (EVs):ti,ab,kw OR (cell derived microparticles):ti,ab,kw OR (microparticle):ti,ab,kw

#2= (vesicles):ti,ab,kw OR (extracellular vesicle):ti,ab,kw OR (exovesicle):ti,ab,kw OR (microvesicle):ti,ab,kw OR (exosome):ti,ab,kw

#3= #1 OR #2

#4=(mesenchymal stem cells):ti,ab,kw OR (Mesenchymal Stromal Cells):ti,ab,kw OR (Msc):ti,ab,kw OR (stem cell):ti,ab,kw

#5= (liver):ti,ab,kw OR (hepatic):ti,ab,kw

#6=(Cirrhotic):ti,ab,kw OR (Cirrhosis):ti,ab,kw OR (Fibrosis):ti,ab,kw OR (Fibrose):ti,ab,kw OR (Disease):ti,ab,kw

#7= (Failure):ti,ab,kw OR (Injure):ti,ab,kw

#8= #6 OR #7

#3 AND #8 AND #9

Table S1：Subgroup analyses of MSC-EV vs. Placebo on ALT result.

	Liver fibrosis		ALI		NAFLD		AIH		I/RI	
	**SMD 95%-CI**	**p-value**	**SMD 95%-CI**	**p-value**	**SMD 95%-CI**	**p-value**	**SMD 95%-CI**	**p-value**	**SMD 95%-CI**	**p-value**
Overall effect	-2.79 [-3.43, -2.15]	<0.01	-3.41 [-4.16, -2.66]	<0.01	-3.53 [-4.62, -2.44]	<0.01	-1.30 [-2.12, -0.49]	<0.01	-3.41 [-4.47, -2.35]	<0.01
Source of MSC										
-BMSC	-4.20[-5.23, -3.17]	<0.01	-4.89 [-6.54, -3.24]	<0.01	-6.81 [-9.32, -4.30]	<0.01	-1.30 [-2.12, -0.49]	<0.01	-3.45 [-4.95, -1.96]	<0.01
-UCMSC	-0.54 [-1.71, 0.62]	0.36	-8.40[-11.62, -5.18]	<0.01	-5.79 [-8.84, -2.75]	<0.01	/	/	-3.37 [-4.87, -1.86]	<0.01
-ADSC	-3.58[-5.95, -1.22]	<0.05	-3.37 [-4.81, -1.93]	<0.01	-2.21 [-3.52, -0.89]	<0.01	/	/	/	/
-ESC	-3.01[-4.69, -1.32]	<0.01	-1.04 [-2.28, 0.20]	0.10	/	/	/	/	/	/
Species										
-mice	-3.74 [-5.99, -1.50]	<0.01	-2.97 [-3.75, -2.19]	<0.01	-2.21 [-3.52, -0.89]	<0.01	-1.30 [-2.12, -0.49]	<0.01	-2.96 [-4.17, -1.76]	<0.01
-rats	-3.62 [-4.56, -2.69]	<0.01	-9.57 [-12.50, -6.65]	<0.01	-6.40 [-8.34, -4.46]	<0.01	/	/	-4.90 [-7.12, -2.69]	<0.01
Administration route										
-i.v.	-4.15 [-5.43, -2.87]	<0.01	-5.00 [-6.15, -3.86]	<0.01	-3.53 [-4.62, -2.44]	<0.01	-0.96 [-1.81, -0.12]	<0.05	-3.41 [-4.47, -2.35]	<0.01
-i.p.	-4.52 [-7.11, -1.94]	<0.01	-4.47 [-6.92, -2.01]	<0.01	/	/	-0.37 [-1.36, 0.62]	0.46	/	/
-i.s.	-2.09 [-4.09, -0.09]	<0.05	-1.04 [-2.28, 0.20]	0.10	/	/	/	/	/	/
Times of treatment										
-single	-2.06 [-2.85, -1.28]	<0.01	-3.41 [-4.16, -2.66]	<0.01	-2.21 [-3.52, -0.89]	<0.01	-11.41 [-17.95, -4.87]	<0.01	-3.41 [-4.47, -2.35]	<0.01
-multiple	-4.24 [-5.40, -3.08]	<0.01	/	/	-6.40 [-8.34, -4.46]	<0.01	-1.14 [-1.96, -0.32]	<0.01	/	/

ALT: alanine aminotransferase; EVs: extracellular vesicles; ALI: acute liver injure; NAFLD: nonalcoholic fatty liver disease; AIH: autoimmune hepatitis; IRI: ischemia-reperfusion injury; MSC: mesenchymal stem cell; BMSC: bone marrow mesenchymal stem cell; UCMSC: umbilical cord mesenchymal stem cell; ADSC: adipose-derived mesenchymal stem cell; ESC: embryonic stem cell; TSC: tonsil-derived mesenchymal stromal cell; MenSC: menstrual blood-derived mesenchymal stem cell; i.v.: intravenous injection; i.p.: intraperitoneal; i.s.: intrasplenic injection

Table S2：Subgroup analyses of MSC-EV vs. Placebo on AST result.

	Liver fibrosis		ALI		NAFLD		AIH		I/RI	
	**SMD 95%-CI**	**p-value**	**SMD 95%-CI**	**p-value**	**SMD 95%-CI**	**p-value**	**SMD 95%-CI**	**p-value**	**SMD 95%-CI**	**p-value**
Overall effect	-2.52 [-3.34, -1.70]	<0.01	-3.36 [-4.09, -2.63]	<0.01	-5.50 [-7.23, -3.77]	<0.01	-1.74 [-2.70, -0.78]	<0.01	-3.72 [-4.88, -2.57]	<0.01
Source of MSC										
-BMSC	-6.51 [-8.77, -4.26]	<0.01	-6.34 [-8.32, -4.36]	<0.01	-6.74 [-9.22, -4.25]	<0.01	-1.74 [-2.70, -0.78]	<0.01	-3.84 [-5.51, -2.17]	<0.01
-UCMSC	-4.09 [-6.38, -1.79]	<0.01	-5.03 [-7.26, -2.79]	<0.01	-4.35 [-6.76, -1.94]	<0.01	/	/	-3.62 [-5.23, -2.01]	<0.01
-ADSC	-2.28 [-4.06, -0.50]	0.01	-3.88 [-5.50, -2.25]	<0.01	/	/	/	/	/	/
-ESC	-0.37 [-1.76, 1.02]	0.60	-1.08 [-2.32, 0.17]	0.09	/	/	/	/	/	/
Species										
-mice	-3.25 [-4.20, -2.29]	<0.01	-3.09 [-3.83, -2.34]	<0.01	/	/	-1.74 [-2.70, -0.78]	<0.01	-3.24 [-4.53, -1.95]	<0.01
-rats	-1.41 [-2.66, -0.16]	<0.05	-9.12 [-12.54, -5.70]	<0.01	-5.50 [-7.23, -3.77]	<0.01	/	/	-5.70 [-8.30, -3.09]	<0.01
Administration route										
-i.v.	-3.62 [-4.75, -2.49]	<0.01	-4.46 [-5.45, -3.48]	<0.01	-6.74 [-9.22, -4.25]	<0.01	-1.42 [-2.40, -0.44]	<0.01	-3.06 [-4.32, -1.81]	<0.01
-i.p.	-0.25 [-1.65, 1.15]	0.73	-4.60 [-7.12, -2.09]	<0.01	/	/	-9.58 [-14.39, -4.77]	<0.01	/	/
-i.s.	/	/	-1.08 [-2.32, 0.17]	0.09	/	/	/	/	/	/
Times of treatment										
-single	-2.39 [-3.30, -1.48]	<0.01	-3.36 [-4.09, -2.63]	<0.01	/	/	-4.12 [-6.75, -1.49]	<0.01	-3.72 [-4.88, -2.57]	<0.01
-multiple	-3.08 [-4.98, -1.18]	<0.01	/	/	-5.50 [-7.23, -3.77]	<0.01	-1.38 [-2.41, -0.34]	<0.01	/	/

AST: aspartate aminotransferase; EVs: extracellular vesicles; ALI: acute liver injure; NAFLD: nonalcoholic fatty liver disease; AIH: autoimmune hepatitis; IRI: ischemia-reperfusion injury; MSC: mesenchymal stem cell; BMSC: bone marrow mesenchymal stem cell; UCMSC: umbilical cord mesenchymal stem cell; ADSC: adipose-derived mesenchymal stem cell; ESC: embryonic stem cell; TSC: tonsil-derived mesenchymal stromal cell; MenSC: menstrual blood-derived mesenchymal stem cell; i.v.: intravenous injection; i.p.: intraperitoneal injection; i.s.: intrasplenic injection

Supplementary tables

Table S3: Results of sensitivity analyses of the effect of MSC-EV therapy vs Placebo on ALB level. (Random-effects model)

	SMD 95%-CI	p-value	*I2*
Pooled estimate	2.31 [0.96, 3.66]	P<0.01	81%
Omitting Rostom 2020	2.58 [1.10, 4.05]	P<0.01	79%
Omitting Sabry 2019	1.96 [0.65, 3.26]	P<0.01	75%
Omitting Takeuchi 2021	2.39 [0.86, 3.92]	P<0.01	83%
Omitting Wang 2021	2.41 [0.93, 3.88]	P<0.01	83%
Omitting Alzahrani 2018	1.81 [0.61, 3.00]	P<0.01	75%
Omitting Damania 2018	2.11 [1.57, 2.66]	P<0.01	79%
Omitting Fang 2021	2.01 [1.45, 2.56]	P<0.01	83%
Omitting Haga 2017	1.99 [1.41, 2.56]	P<0.01	83%
Omitting Damania 2018	1.93 [1.39, 2.47]	P<0.01	81%
Omitting Watanabe 2020	2.10 [1.50, 2.71]	P<0.01	83

Table S4: Results of sensitivity analyses of the effect of MSC-EV therapy vs Placebo on ALT level. (Random-effects model)

	SMD 95%-CI	p-value	*I2*
Pooled estimate	-2.79 [-3.16, -2.43]	P<0.01	76%
Omitting Angioni 2020	-2.78 [-3.15, -2.41]	P<0.01	77%
Omitting Kim 2021	-2.78 [-3.15, -2.41]	P<0.01	77%
Omitting Li 2013	-3.05 [-3.44, -2.67]	P<0.01	74%
Omitting Mardpour 2018	-2.83 [-3.20, -2.47]	P<0.01	76%
Omitting Mardpour 2019	-2.79 [-3.15, -2.42]	P<0.01	76%
Omitting Rong 2019	-2.80 [-3.16, -2.43]	P<0.01	76%
Omitting Rostom 2020	-2.81 [-3.18, -2.44]	P<0.01	76%
Omitting Sabry 2019	-2.69 [-3.06, -2.31]	P<0.01	75%
Omitting Takeuchi 2021	-2.79 [-3.16, -2.42]	P<0.01	76%
Omitting Wang 2021	-2.80 [-3.16, -2.43]	P<0.01	76%
Omitting Xuan 2022	-2.77 [-3.14, -2.41]	P<0.01	75%
Omitting Alzahrani 2018	-2.75 [-3.11, -2.38]	P<0.01	74%
Omitting Chen 2017	-2.72 [-3.09, -2.36]	P<0.01	75%
Omitting Fang 2021	-2.85 [-3.22, -2.48]	P<0.01	76%
Omitting Haga 2017	-2.77 [-3.14, -2.41]	P<0.01	76%
Omitting Liu 2018	-2.73 [-3.10, -2.36]	P<0.01	76%
Omitting Tan 2014	-2.97 [-3.35, -2.60]	P<0.01	75%
Omitting Yan 2017	-2.75 [-3.11, -2.38]	P<0.01	76%
Omitting Zhang 2022	-2.82 [-3.18, -2.45]	P<0.01	76%
Omitting Zhang2 2020	-2.80 [-3.16, -2.44]	P<0.01	76%
Omitting Cheng 2021	-2.77 [-3.13, -2.40]	P<0.01	76%
Omitting E-Derany 2021	-2.72 [-3.09, -2.36]	P<0.01	75%
Omitting Watanabe 2020	-2.86 [-3.24, -2.48]	P<0.01	76%
Omitting Chen 2018	-3.19 [-3.58, -2.80]	P<0.01	71%
Omitting Lu 2019	-2.77 [-3.13, -2.40]	P<0.01	76%
Omitting Tamura 2016	-2.85 [-3.22, -2.48]	P<0.01	76%
Omitting Zhao 2021	-2.78 [-3.15, -2.42]	P<0.01	75%
Omitting Anger 2019	-2.84 [-3.21, -2.46]	P<0.01	76%
Omitting Damania 2018	-2.78 [-3.14, -2.42]	P<0.01	76%
Omitting Haga2 2017	-2.76 [-3.12, -2.39]	P<0.01	76%
Omitting Yao 2019	-2.77 [-3.14, -2.41]	P<0.01	77%
Omitting Zhang 2020	-2.79 [-3.15, -2.42]	P<0.01	77%
Omitting Zheng 2020	-2.79 [-3.15, -2.42]	P<0.01	77%

Table S5: Results of sensitivity analyses of the effect of MSC-EV therapy vs Placebo on AST level. (Random-effects model)

	SMD 95%-CI	p-value	*I2*
Pooled estimate	-2.99 [-3.42, -2.57]	P<0.01	75%
Omitting Kim 2021	-3.00 [-3.44, -2.56]	P<0.01	75%
Omitting Li 2013	-2.95 [-3.37, -2.53]	P<0.01	75%
Omitting Mardpour 2019	-3.25 [-3.69, -2.82]	P<0.01	71%
Omitting Rong 2019	-2.97 [-3.39, -2.56]	P<0.01	75%
Omitting Rostom 2020	-2.93 [-3.35, -2.51]	P<0.01	74%
Omitting Wang 2021	-2.98 [-3.39, -2.56]	P<0.01	75%
Omitting Xuan 2022	-2.93 [-3.35, -2.52]	P<0.01	74%
Omitting You 2021	-3.03 [-3.46, -2.60]	P<0.01	75%
Omitting Alzahrani 2018	-2.91 [-3.33, -2.49]	P<0.01	71%
Omitting Chen 2017	-2.93 [-3.36, -2.50]	P<0.01	75%
Omitting Damania 2018	-2.95 [-3.37, -2.54]	P<0.01	75%
Omitting Fang 2021	-3.04 [-3.46, -2.61]	P<0.01	75%
Omitting Haga 2017	-2.94 [-3.36, -2.52]	P<0.01	75%
Omitting Liu 2018	-2.88 [-3.30, -2.46]	P<0.01	74%
Omitting Tan 2014	-3.23 [-3.67, -2.79]	P<0.01	73%
Omitting Yan 2017	-2.91 [-3.34, -2.49]	P<0.01	75%
Omitting Zhang 2022	-2.97 [-3.39, -2.56]	P<0.01	75%
Omitting Cheng 2021	-2.95 [-3.37, -2.52]	P<0.01	75%
Omitting E-Derany 2021	-2.88 [-3.30, -2.46]	P<0.01	73%
Omitting Anger 2019	-3.01 [-3.43, -2.58]	P<0.01	75%
Omitting Haga2 2017	-2.93 [-3.35, -2.51]	P<0.01	74%
Omitting Yao 2019	-2.95 [-3.37, -2.53]	P<0.01	75%
Omitting Zhang 2020	-2.97 [-3.39, -2.56]	P<0.01	75%
Omitting Chen 2018	-3.36 [-3.81, -2.90]	P<0.01	71%
Omitting Lu 2019	-2.94 [-3.36, -2.52]	P<0.01	74%
Omitting Zhao 2021	-2.96 [-3.38, -2.54]	P<0.01	75%
Omitting Zheng 2020	-3.00 [-3.42, -2.57]	P<0.01	75%

Table S6: Results of sensitivity analyses of the effect of MSC-EV therapy vs Placebo on damage area. (Random-effects model)

	SMD 95%-CI	p-value	*I2*
Pooled estimate	-3.73 [-4.74, -2.73]	P<0.01	61%
Omitting Alhomrani 2017	-3.67 [-4.69, -2.65]	P<0.01	62%
Omitting Gupta 2021	-3.78 [-4.91, -2.66]	P<0.01	64%
Omitting Han 2020	-3.90 [-5.00, -2.81]	P<0.01	64%
Omitting Mardpour 2018	-3.56 [-4.51, -2.60]	P<0.01	58%
Omitting Mardpour 2019	-3.57 [-4.55, -2.59]	P<0.01	60%
Omitting Ohara 2018	-4.05 [-5.17, -2.93]	P<0.01	59%
Omitting Rong 2019	-3.68 [-4.69, -2.66]	P<0.01	62%
Omitting Rostom 2020	-3.96 [-5.08, -2.85]	P<0.01	63%
Omitting Takeuchi 2021	-3.92 [-5.01, -2.82]	P<0.01	64%
Omitting Xuan 2022	-3.43 [-4.36, -2.49]	P<0.01	55%
Omitting You 2021	-3.84 [-4.93, -2.76]	P<0.01	63%
Omitting Watanabe 2020	-4.03 [-5.13, -2.94]	P<0.01	59%
Omitting Anger 2019	-4.17 [-5.36, -2.97]	P<0.01	62%
Omitting Haga2 2017	-3.70 [-4.73, -2.68]	P<0.01	53%
Omitting Yao 2019	-3.87 [-4.97, -2.77]	P<0.01	58%

Table S7: Results of sensitivity analyses of the effect of MSC-EV therapy vs Placebo on TNF-α (Random-effects model)

	SMD 95%-CI	p-value	*I2*
Pooled estimate	-4.60 [-6.45, -2.75]	P<0.01	79%
Omitting Mardpour 2018	-4.27 [-6.06, -2.48]	P<0.01	79%
Omitting Mardpour 2019	-4.68 [-6.71, -2.65]	P<0.01	81%
Omitting Chen 2017	-3.58 [-5.00, -2.16]	P<0.01	64%
Omitting Liu 2018	-4.43 [-6.41, -2.46]	P<0.01	80%
Omitting Yan 2017	-5.17 [-7.32, -3.03]	P<0.01	79%
Omitting Chen 2018	-4.39 [-6.35, -2.42]	P<0.01	79%
Omitting Zhao 2021	-5.11 [-7.30, -2.92]	P<0.01	81%
Omitting Yao 2019	-4.88 [-7.02, -2.75]	P<0.01	81%
Omitting Zheng 2020	-5.16 [-7.33, -2.99]	P<0.01	80%

Table S8: Results of sensitivity analyses of the effect of MSC-EV therapy vs Placebo on IL-1β. (Random-effects model)

	**SMD 95%-CI**	**p-value**	***I2***
Pooled estimate	-4.34 [-6.02, -2.66]	P<0.01	77%
Omitting Chen 2017	-3.42 [-4.67, -2.16]	P<0.01	59%
Omitting Jiang 2019	-4.87 [-6.67, -3.06]	P<0.01	72%
Omitting Liu 2018	-4.58 [-6.77, -2.40]	P<0.01	81%
Omitting Zhang2 2020	-4.20 [-5.84, -2.56]	P<0.01	78%
Omitting Yao 2019	-4.30 [-6.12, -2.47]	P<0.01	79%
Omitting Chen 2018	-4.73 [-6.79, -2.67]	P<0.01	80%
Omitting Lu 2019	-4.13 [-5.89, -2.37]	P<0.01	77%
Omitting Zhao 2021	-4.76 [-6.73, -2.78]	P<0.01	79%

Table S9: Results of sensitivity analyses of the effect of MSC-EV therapy vs Placebo on IL-6. (Random-effects model)

	**SMD 95%-CI**	**p-value**	***I2***
Pooled estimate	-5.26 [-7.07, -3.45]	P<0.01	70%
Chen 2017	-4.57 [-6.20, -2.93]	P<0.01	59%
Jiang 2019	-4.78 [-6.51, -3.05]	P<0.01	66%
Liu 2018	-5.04 [-6.94, -3.13]	P<0.01	71%
Yan 2017	-5.45 [-7.52, -3.38]	P<0.01	74%
Zhang2 2020	-5.10 [-6.92, -3.29]	P<0.01	72%
Lu 2019	-5.70 [-7.86, -3.53]	P<0.01	73%
Zhao 2021	-5.81 [-7.76, -3.87]	P<0.01	65%
Yao 2019	-5.21 [-7.18, -3.24]	P<0.01	73%
Zheng 2020	-5.77 [-7.87, -3.67]	P<0.01	71%

ALT: alanine aminotransferase; EVs: extracellular vesicles; ALI: acute liver injure; NAFLD: nonalcoholic fatty liver disease; AIH: autoimmune hepatitis; IRI: ischemi

Table S10: CAMARADEA quality assessment

Study (year)	1	2	3	4	5	6	7	8	9	10	score
Xuan et al. (2022) [31]					✓	✓		✓	✓	✓	5
Zhang et al. (2022) [42]					✓	✓	✓	✓	✓		5
Wang et al. (2021) [30]					✓	✓		✓	✓	✓	5
Kim et al. (2021) [22]		✓			✓	✓		✓	✓	✓	6
Zhao et al. (2021) [49]					✓	✓		✓	✓		4
Fang et al. (2021) [36]		✓			✓	✓	✓	✓	✓	✓	7
You et al. (2021) [32]					✓	✓		✓	✓	✓	5
Cheng et al. (2021) [44]		✓			✓	✓	✓	✓	✓	✓	7
E-Derany et al. (2021) [45]		✓			✓	✓	✓	✓	✓	✓	7
Gupta et al. (2021) [20]		✓			✓	✓		✓	✓	✓	6
Takeuchi et al. (2021) [29]					✓	✓	✓	✓	✓	✓	6
Rostom et al. (2020) [27]					✓	✓		✓	✓	✓	5
Han et al. (2020) [21]					✓	✓		✓	✓	✓	5
Zheng et al. (2020) [54]		✓			✓	✓	✓	✓	✓	✓	7
Zhang1 et al. (2020) [53]		✓			✓	✓	✓	✓	✓	✓	7
Angioni et al. (2020) [19]					✓	✓		✓	✓	✓	5
Zhang2 et al. (2020) [43]					✓	✓	✓	✓	✓	✓	6
Watanabe et al. (2020) [46]					✓	✓		✓	✓	✓	5
Rong et al. (2019) [26]		✓			✓	✓		✓	✓	✓	6
Jiang et al. (2019) [38]		✓			✓	✓	✓	✓	✓		6
Lu et al. (2019) [48]		✓			✓	✓		✓	✓	✓	6
Anger et al. (2019) [50]					✓	✓	✓	✓	✓		5
Yao et al. (2019) [52]		✓			✓	✓			✓	✓	5
Mardpour et al. (2019) [24]					✓	✓		✓	✓	✓	5
Ohara et al. (2018) [25]					✓	✓	✓	✓	✓	✓	6
Mardpour et al. (2018) [23]					✓	✓	✓	✓	✓	✓	6
Chen et al. (2018) [47]					✓	✓			✓	✓	4
Liu et al. (2018) [39]					✓	✓		✓	✓	✓	5
Chen et al. (2017) [34]					✓	✓		✓	✓	✓	5
Yan et al. (2017) [41]		✓			✓	✓		✓	✓	✓	6
Li et al. (2013) [14]					✓	✓		✓	✓	✓	5
Tamura et al. (2016) [15]					✓	✓		✓	✓	✓	5
Tan et al. (2014) [40]					✓	✓	✓	✓	✓	✓	6
Haga2 et al. (2017) [51]					✓	✓		✓	✓	✓	5
Alhomrani et al. (2017) [18]					✓	✓		✓	✓		4
Sabry et al. (2019) [28]					✓	✓		✓	✓	✓	5
Haga et al. (2017) [37]					✓	✓		✓	✓	✓	5
Alzahrani et al. (2018) [33]		✓			✓	✓	✓	✓	✓	✓	7
Damania et al. (2018) [35]					✓	✓	✓	✓	✓	✓	6

Abbreviation: 1. calculation of sample size; 2. animals were randomly allocated; 3. blinded model ;4. blinded outcome assessment; 5. appropriate animal model; 6. use of anesthetic without significant

protective or toxic effects on the liver; 7. temperature control; 8. statement of compliance with animal welfare regulations; 9. peer-reviewed journal; and 10. statement of potential conflict of interests.

Supplementary figures

Results of meta-analyses

Study or Subgroup	Experimental Mean	Experimental SD	Experimental Total	Control Mean	Control SD	Control Total	Weight	Std. Mean Difference IV, Random, 95% CI
**1.2.1 liver fibrosis**								
Kim 2021	32.31	9.84	6	80.31	20	5	4.5%	-2.88 [-4.80, -0.97]
Li 2013	115.85	14.63	6	167.07	7.32	6	4.1%	-4.09 [-6.38, -1.79]
Mardpour 2019	124.28	288.57	4	182.85	21.43	4	4.9%	-0.25 [-1.65, 1.15]
Rong 2019	170.49	39.34	3	603.28	76.69	3	1.9%	-5.68 [-11.40, 0.03]
Rostom 2020	105.34	12.57	6	250.2	28.74	6	3.4%	-6.03 [-9.18, -2.88]
Wang 2021	325.93	14.82	3	627.16	14.82	3	0.3%	-16.26 [-32.04, -0.48]
Xuan 2022	297.55	18.64	6	506.94	30.51	6	2.9%	-7.65 [-11.55, -3.75]
You 2021	381.93	41.29	5	939.35	309.67	5	4.6%	-2.28 [-4.06, -0.50]
**Subtotal (95% CI)**			**39**			**38**	**26.5%**	**-3.79 [-5.69, -1.90]**
Heterogeneity: Tau² = 4.62; Chi² = 27.64, df = 7 (P = 0.0003); I² = 75%								
Test for overall effect: Z = 3.92 (P < 0.0001)								
**1.2.2 acute liver injury**								
Alzahrani 2018	73.6	1.99	9	105.4	3.04	9	2.5%	-11.79 [-16.24, -7.33]
Chen 2017	234.13	23.81	10	369.05	41.66	10	4.7%	-3.81 [-5.39, -2.23]
Damania 2018	150	7.69	3	315.38	34.61	3	2.0%	-5.28 [-10.62, 0.06]
Fang 2021	1,905.26	126.31	3	2,915.79	694.74	3	4.2%	-1.62 [-3.86, 0.62]
Haga 2017	262.201	92.46	6	1,873.68	447.49	6	4.0%	-4.60 [-7.12, -2.09]
Liu 2018	420.15	106.37	6	1,156.61	127.27	6	3.5%	-5.80 [-8.84, -2.75]
Liu 2018	257.9	73.68	6	994.73	110.52	6	3.0%	-7.24 [-10.95, -3.53]
Tan 2014	1,148.51	792.08	6	2,534.65	1,485.15	6	5.0%	-1.08 [-2.32, 0.17]
Yan 2017	75.47	7.54	5	415.09	101.88	5	3.8%	-4.25 [-6.94, -1.55]
Yan 2017	50.31	10.06	5	347.17	55.34	5	2.8%	-6.74 [-10.73, -2.75]
Zhang 2022	19.15	6.38	3	191.48	19.15	3	0.9%	-9.66 [-19.12, -0.20]
**Subtotal (95% CI)**			**62**			**62**	**36.4%**	**-4.88 [-6.60, -3.15]**
Heterogeneity: Tau² = 5.54; Chi² = 42.24, df = 10 (P < 0.00001); I² = 76%								
Test for overall effect: Z = 5.54 (P < 0.00001)								
**1.2.3 nonalcoholic fatty liver disease**								
Cheng 2021	50.48	4.85	6	75.73	5.82	6	4.0%	-4.35 [-6.76, -1.94]
E-Derany 2021	52	0.4	10	71	3.8	10	4.0%	-6.74 [-9.22, -4.25]
**Subtotal (95% CI)**			**16**			**16**	**8.0%**	**-5.52 [-7.86, -3.18]**
Heterogeneity: Tau² = 1.28; Chi² = 1.82, df = 1 (P = 0.18); I² = 45%								
Test for overall effect: Z = 4.63 (P < 0.00001)								
**1.2.5 Ischemia reperfusion injury**								
Anger 2019	3,788.72	757.14	5	5,726.32	590.24	5	4.5%	-2.58 [-4.49, -0.67]
Damania 2018	217.24	6.89	5	341.37	6.89	3	0.7%	-15.67 [-26.54, -4.80]
Haga2 2017	753.63	165.04	6	6,094.5	962.27	6	3.0%	-7.14 [-10.81, -3.48]
Yao 2019	533.72	121.13	5	1,271.67	151.51	5	3.5%	-4.86 [-7.86, -1.86]
Zhang 2020	1,622.85	137.14	3	2,834.29	182.85	3	1.7%	-6.00 [-12.00, 0.01]
Zheng 2020	1,408.28	331.07	5	2,520.95	384.39	5	4.4%	-2.80 [-4.81, -0.80]
**Subtotal (95% CI)**			**29**			**27**	**17.9%**	**-4.53 [-6.55, -2.51]**
Heterogeneity: Tau² = 3.05; Chi² = 11.27, df = 5 (P = 0.05); I² = 56%								
Test for overall effect: Z = 4.39 (P < 0.0001)								
**1.2.6 autoimmune hepatitis**								
Chen 2018	302.29	75.56	8	440.97	173.27	8	5.1%	-0.98 [-2.04, 0.07]
Lu 2019	420	30	6	772.5	37.5	6	2.3%	-9.58 [-14.39, -4.77]
Zhao 2021	850.47	56.07	5	1,654.21	242.99	5	3.9%	-4.12 [-6.75, -1.49]
**Subtotal (95% CI)**			**19**			**19**	**11.2%**	**-4.30 [-8.39, -0.21]**
Heterogeneity: Tau² = 10.77; Chi² = 15.33, df = 2 (P = 0.0005); I² = 87%								
Test for overall effect: Z = 2.06 (P = 0.04)								
**Total (95% CI)**			**165**			**162**	**100.0%**	**-4.45 [-5.42, -3.49]**
Heterogeneity: Tau² = 4.45; Chi² = 116.72, df = 29 (P < 0.00001); I² = 75%								
Test for overall effect: Z = 9.07 (P < 0.00001)								
Test for subgroup differences: Chi² = 1.42, df = 4 (P = 0.84), I² = 0%								

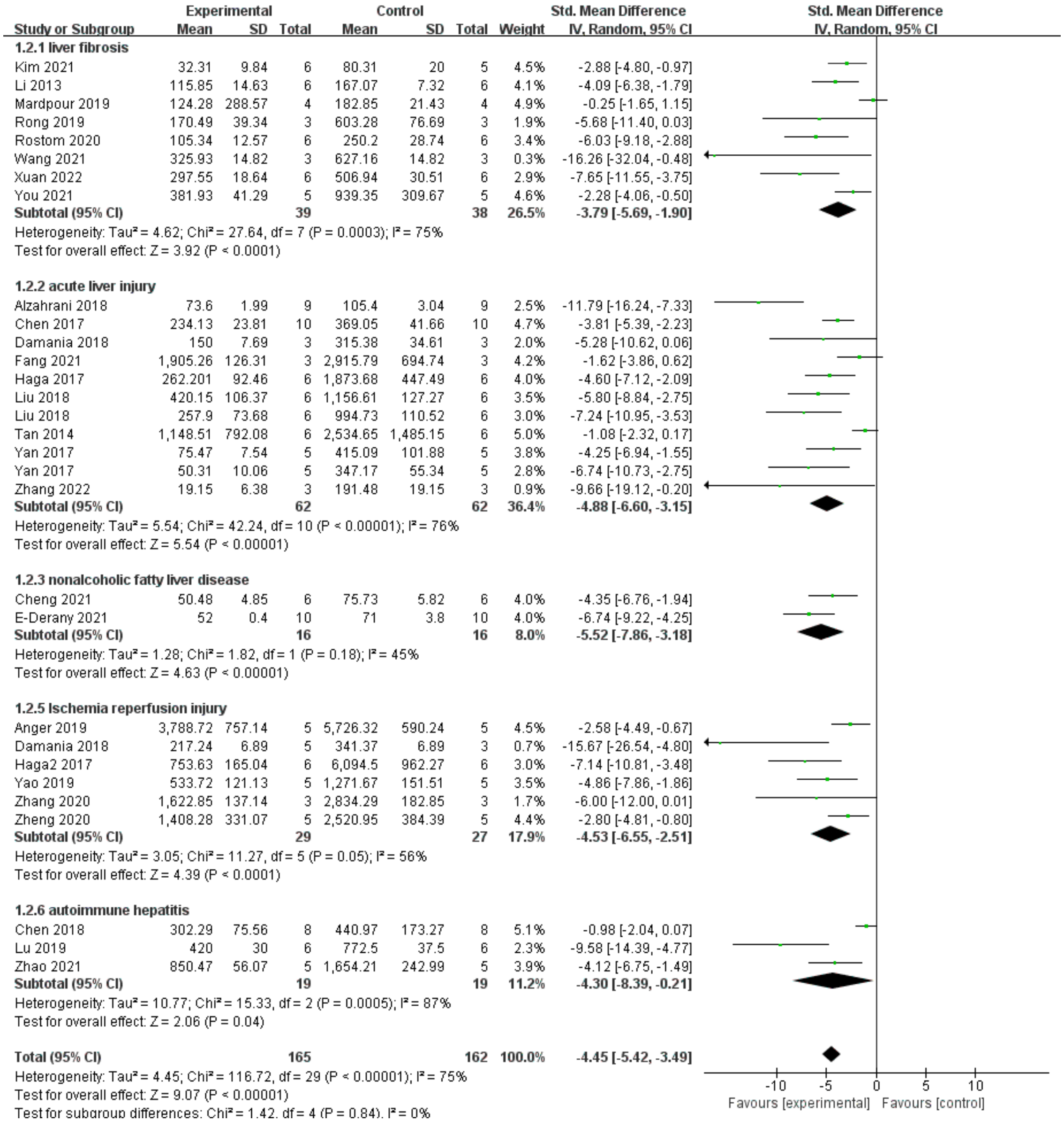

Figure S1: Forest plot of the efficacy of MSC-EV therapy on aspartate aminotransferase (AST).

Study or Subgroup	Experimental Mean	Experimental SD	Experimental Total	Control Mean	Control SD	Control Total	Weight	Std. Mean Difference IV, Random, 95% CI
**1.3.1 liver fibrosis**								
Angioni 2020	386.21	68.96	4	682.75	62.07	3	8.9%	-3.77 [-7.11, -0.43]
Mardpour 2018	535.14	97.3	4	867.57	162.16	4	13.9%	-2.16 [-4.19, -0.13]
Mardpour 2019	473.14	171.43	4	874.92	171.48	4	14.1%	-2.04 [-4.01, -0.07]
Rong 2019	97.61	9.78	3	297.37	55.28	3	6.6%	-4.03 [-8.23, 0.18]
Takeuchi 2021	214	11.48	5	248.64	13.38	5	14.6%	-2.51 [-4.39, -0.63]
**Subtotal (95% CI)**			**20**			**19**	**58.1%**	**-2.50 [-3.54, -1.46]**
Heterogeneity: Tau² = 0.00; Chi² = 1.38, df = 4 (P = 0.85); I² = 0%								
Test for overall effect: Z = 4.73 (P < 0.00001)								
**1.3.2 acute liver injure**								
Alzahrani 2018	146.7	3.03	9	200.7	6.32	9	7.2%	-10.38 [-14.32, -6.43]
Haga 2017	91.87	4.37	6	114.13	3.5	6	10.8%	-5.19 [-7.97, -2.42]
**Subtotal (95% CI)**			**15**			**15**	**18.0%**	**-7.59 [-12.65, -2.52]**
Heterogeneity: Tau² = 10.42; Chi² = 4.44, df = 1 (P = 0.04); I² = 77%								
Test for overall effect: Z = 2.93 (P = 0.003)								
**1.3.3 Ischemia reperfusion injury**								
Haga2 2017	93.8	9.2	6	190.5	31.26	6	13.1%	-3.87 [-6.08, -1.67]
Yao 2019	70.86	10.76	5	139.47	16.87	5	10.8%	-4.38 [-7.14, -1.62]
**Subtotal (95% CI)**			**11**			**11**	**23.9%**	**-4.07 [-5.79, -2.35]**
Heterogeneity: Tau² = 0.00; Chi² = 0.08, df = 1 (P = 0.78); I² = 0%								
Test for overall effect: Z = 4.63 (P < 0.00001)								
**Total (95% CI)**			**46**			**45**	**100.0%**	**-3.85 [-5.16, -2.53]**
Heterogeneity: Tau² = 2.18; Chi² = 18.52, df = 8 (P = 0.02); I² = 57%								
Test for overall effect: Z = 5.73 (P < 0.00001)								
Test for subgroup differences: Chi² = 5.50, df = 2 (P = 0.06), I² = 63.6%								

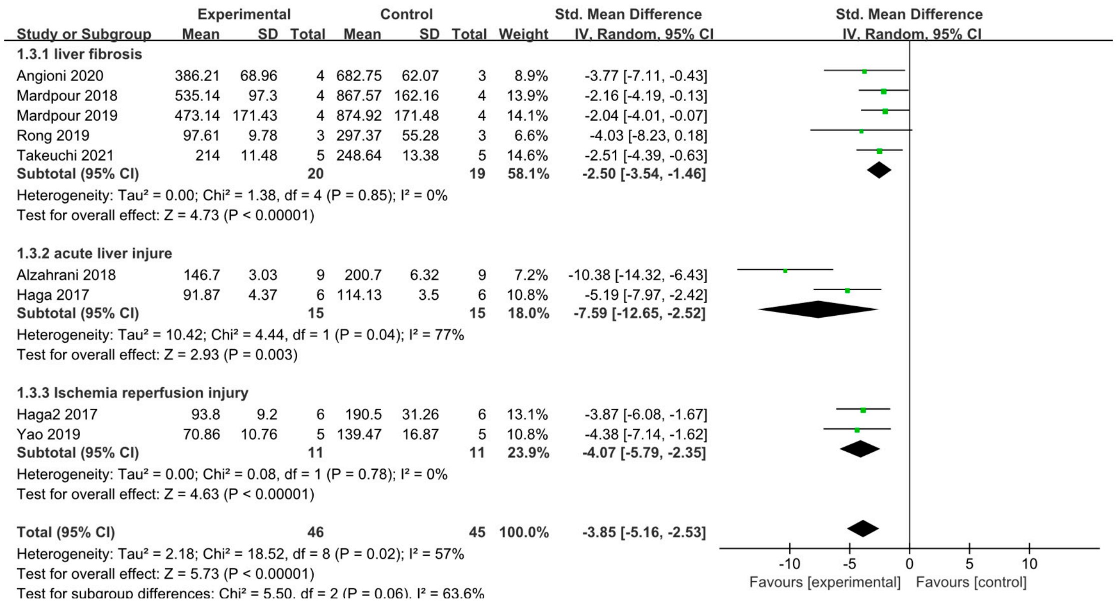

Figure S2: Forest plot of the efficacy of MSC-EV therapy on alkaline phosphatase (ALP).

Study or Subgroup	Experimental Mean	Experimental SD	Experimental Total	Control Mean	Control SD	Control Total	Weight	Std. Mean Difference IV, Fixed, 95% CI
**1.4.1 liver fibrosis**								
Mardpour 2018	14.89	2.58	4	29.53	7.23	4	38.2%	-2.35 [-4.47, -0.22]
Mardpour 2019	5.71	2.28	4	29.4	7.31	4	19.7%	-3.80 [-6.77, -0.84]
Rong 2019	3.72	0.84	3	10.08	2.16	3	15.0%	-3.10 [-6.50, 0.29]
**Subtotal (95% CI)**			**11**			**11**	**72.9%**	**-2.90 [-4.44, -1.36]**
Heterogeneity: Chi² = 0.63, df = 2 (P = 0.73); I² = 0%								
Test for overall effect: Z = 3.69 (P = 0.0002)								
**1.4.2 acute liver injure**								
Fang 2021	10.99	0.53	3	13.77	1.46	3	27.1%	-2.02 [-4.55, 0.50]
**Subtotal (95% CI)**			**3**			**3**	**27.1%**	**-2.02 [-4.55, 0.50]**
Heterogeneity: Not applicable								
Test for overall effect: Z = 1.57 (P = 0.12)								
**Total (95% CI)**			**14**			**14**	**100.0%**	**-2.66 [-3.97, -1.35]**
Heterogeneity: Chi² = 0.97, df = 3 (P = 0.81); I² = 0%								
Test for overall effect: Z = 3.96 (P < 0.0001)								
Test for subgroup differences: Chi² = 0.33, df = 1 (P = 0.56), I² = 0%								

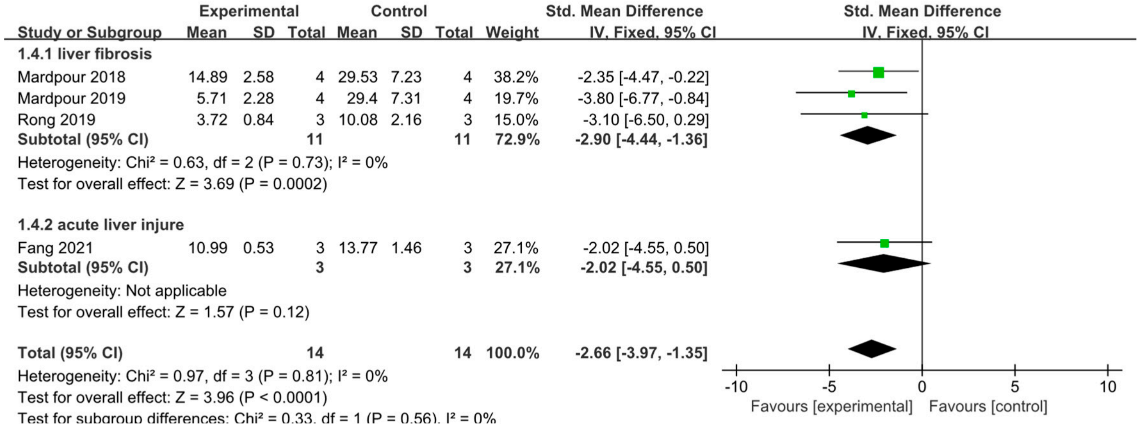

Figure S3: Forest plot of the efficacy of MSC-EV therapy on γ-glutamyl transferase (γ-GT).

	Experimental			Control				Std. Mean Difference
Study or Subgroup	Mean	SD	Total	Mean	SD	Total	Weight	IV, Random, 95% CI
**1.11.1 liver fibrosis**								
Gupta 2021	6.7	0.7	5	5.2	0.3	5	13.3%	2.52 [0.63, 4.40]
Gupta 2021	6.9	0.2	5	5.2	0.3	5	9.2%	6.02 [2.41, 9.63]
Han 2020	1.16	0.04	5	1.48	0.08	5	10.9%	-4.57 [-7.43, -1.71]
Kim 2021	5.6	0.2	5	5.5	0.4	6	14.8%	0.28 [-0.92, 1.48]
Rong 2019	3.05	0.11	3	4.47	0.08	3	1.9%	-11.81 [-23.33, -0.29]
**Subtotal (95% CI)**			**23**			**24**	**50.1%**	**0.19 [-3.02, 3.39]**
Heterogeneity: Tau² = 9.77; Chi² = 29.97, df = 4 (P < 0.00001); I² = 87%								
Test for overall effect: Z = 0.11 (P = 0.91)								
**1.11.2 acute liver injure**								
Fang 2021	6.2	0.2	3	6.7	0.1	3	10.8%	-2.53 [-5.45, 0.39]
Zhang2 2020	4.37	0.07	3	4.68	0.1	3	10.1%	-2.87 [-6.08, 0.33]
**Subtotal (95% CI)**			**6**			**6**	**20.8%**	**-2.69 [-4.84, -0.53]**
Heterogeneity: Tau² = 0.00; Chi² = 0.02, df = 1 (P = 0.88); I² = 0%								
Test for overall effect: Z = 2.44 (P = 0.01)								
**1.11.3 nonalcoholic fatty liver disease**								
Cheng 2021	3.69	0.31	6	4.7	0.51	6	14.1%	-2.21 [-3.77, -0.65]
Watanabe 2020	7.63	0.67	8	8.57	1.07	8	15.1%	-1.00 [-2.05, 0.06]
**Subtotal (95% CI)**			**14**			**14**	**29.1%**	**-1.46 [-2.62, -0.30]**
Heterogeneity: Tau² = 0.27; Chi² = 1.59, df = 1 (P = 0.21); I² = 37%								
Test for overall effect: Z = 2.48 (P = 0.01)								
**Total (95% CI)**			**43**			**44**	**100.0%**	**-0.81 [-2.48, 0.86]**
Heterogeneity: Tau² = 4.51; Chi² = 44.82, df = 8 (P < 0.00001); I² = 82%								
Test for overall effect: Z = 0.95 (P = 0.34)								
Test for subgroup differences: Chi² = 2.22, df = 2 (P = 0.33), I² = 9.8%								

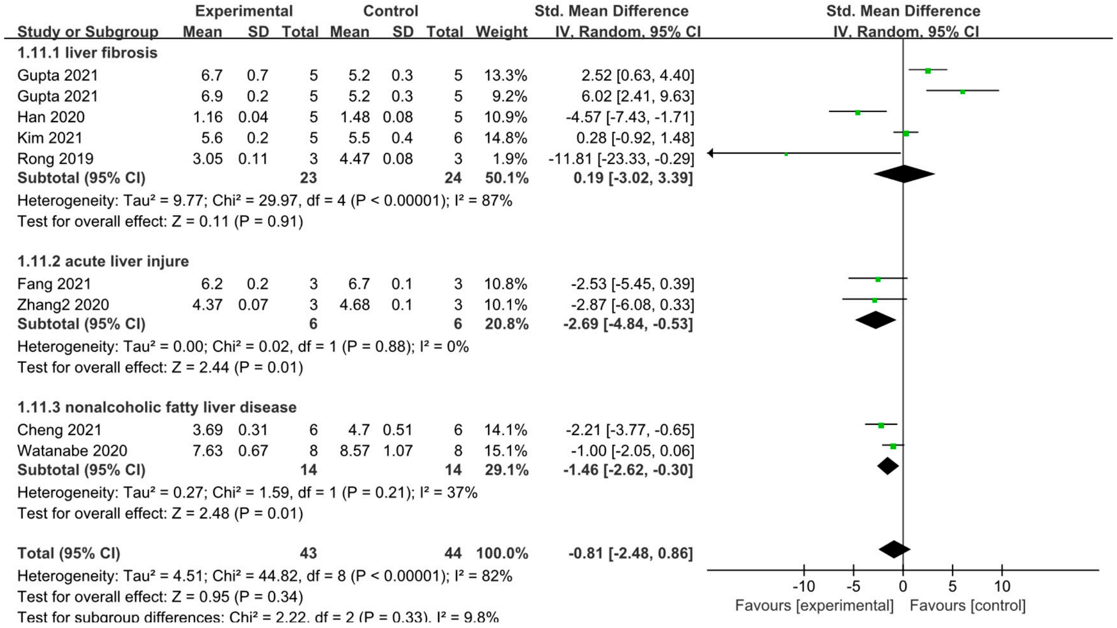

Figure S4: Forest plot of the efficacy of MSC-EV therapy on liver index.

Results of visualized publication bias

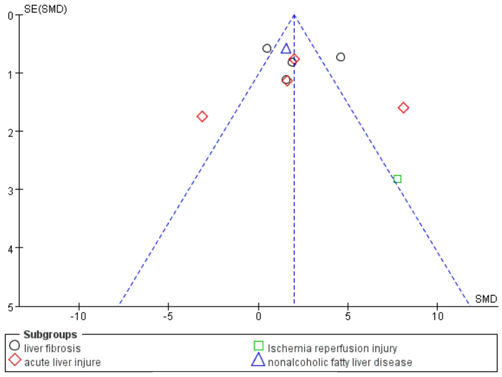

A. ALB

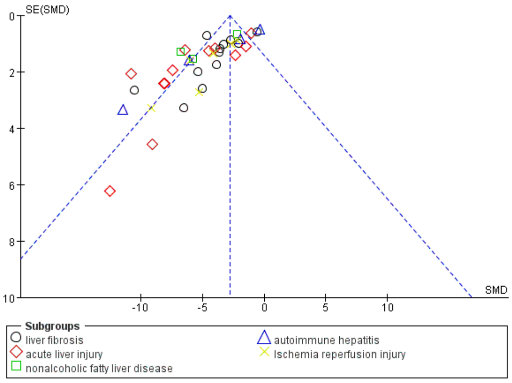

B. ALT

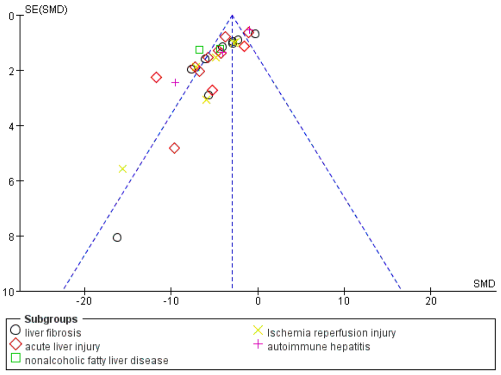

C. AST

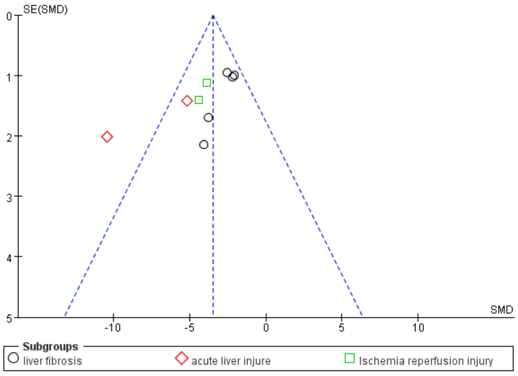

D. ALP

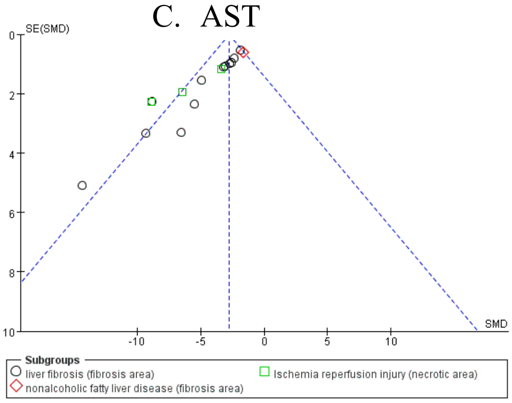

E. Injure area

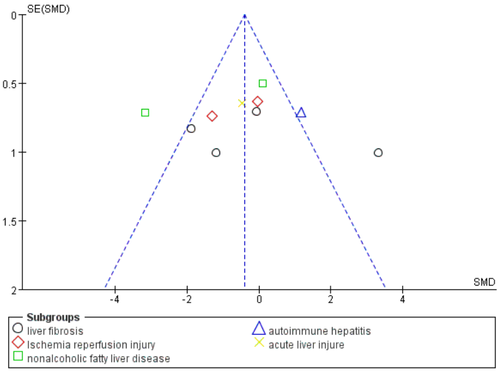

F. ALT

Figure S5: Funnel pots for ALB, ALT, AST, ALP and damage area on MSC-EVs vs Placebo (A-E); ALT on MSC-EVs vs MSC (F).
